# Symmetry-Induced Topological Obstructions in Woodward–Hoffmann Forbidden Reactions: A Molecular Qubit Representation

**DOI:** 10.1021/acs.jpca.6c04534

**Published:** 2026-09-15

**Authors:** Subha Sandeep Repaka, Thillaiarasi Sukumar, Bhagyashri Devaru Bhat, Ran Friedman, Baswanth Oruganti

**Affiliations:** † Department of Foundational Sciences (Mathematics), Ramaiah Institute of Science and Management, Bengaluru 560064, India; ‡ Department of Chemistry, 673783 SRM University-AP, Amaravati 522240, India; § School of Mathematics and Natural Sciences, Chanakya University, Bengaluru 562165, India; ∥ Department of Chemistry and Biomedical Sciences, 4180 Linnaeus University, Kalmar SE-39182, Sweden; ⊥ Department of Mathematics and Mathematical Statistics, Umeå University, Umeå SE-90187, Sweden

## Abstract

Woodward–Hoffmann (WH) rules for electrocyclic reactions remain to date one of the most successful predictive tools in theoretical organic chemistry. At the heart of these rules is the simple idea that preserving bonding continuity in the highest occupied molecular orbital (HOMO) confers a kinetic advantage compared to a pathway that involves bonding disruption. However, this interpretation has been challenged by a few examples of symmetry-forbidden reactions that exhibit similar or lower kinetic barriers than corresponding allowed pathways. In this study, we develop a generalization of the WH rules through two complementary group-theoretic formulations: the Unitary Evolution Inaccessibility Theorem (UEIT) and the Topological Obstruction to Bonding Disruption Theorem (TOBDT). UEIT establishes that symmetry-forbidden reactions correspond to dynamical inaccessibility between the initial and target states under symmetry-preserving unitary evolution, while the TOBDT demonstrates that disruption in bonding continuity in a forbidden reaction constitutes a symmetry-induced topological obstruction. Within the frontier orbital subspace, the TOBDT is realized through a molecular qubit framework in which the orbital mixing angle Θ serves as a geometric descriptor of topological obstruction. This framework is verified through DFT and CASSCF calculations on four prototype 6π-electrocyclization reactions spanning diradicaloid and zwitterionic classes of transition states (TSs). Our results demonstrate that ΔΘ—the change in Θ between the reactant and the TS—correctly distinguishes diradicaloid from zwitterionic pathways. Additionally, computed nucleus-independent chemical shift (NICS_*zz*_) profiles confirm the antiaromatic character of diradicaloid TSs and the nonaromatic character of zwitterionic TSs, suggesting that the orbital symmetry restrictions may not be applicable in the latter.

## Graphical abstract

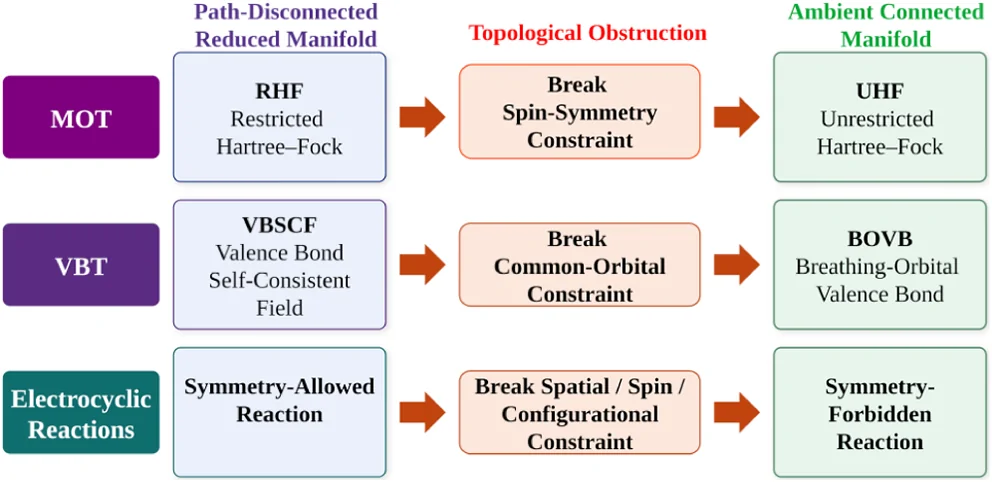

## Introduction

Understanding and predicting how the symmetry of molecular orbitals (MOs) governs reaction pathways and the associated stereochemical outcomes of concerted pericyclic reactions was one of the major milestones in theoretical organic chemistry, pioneered by Woodward and Hoffmann (WH) in the 1960s.^1,2^ In their celebrated 1965 paper,^1^ they demonstrated that the stereochemistry of electrocyclic reactions of polyenes is determined by the symmetry of the highest occupied molecular orbital (HOMO) in the open-ring isomer.^1^ Specifically, in the electronic ground state of an open-ring isomer of a polyene with 4*n*π-electrons, the symmetry of HOMO is such that a conrotatory displacement of the terminal orbital lobes is required to maintain phase continuity, i.e., a bonding interaction,^1^ as illustrated in Scheme 1. Conversely, a disrotatory displacement is required to ensure phase continuity in systems with (4*n*+2)­π-electrons.^1,3^ In photochemical electrocyclic reactions, electronic excitation to the lowest unoccupied molecular orbital (LUMO) reverses phase relationships at the terminal atoms, making 4*n*π-cyclizations disrotatory and (4*n*+2)­π-cyclizations conrotatory, as depicted in Scheme 1. Reactions that do not follow these rules are termed forbidden because antibonding interactions between the overlapping lobes raise the energy of the system along the reaction coordinate, resulting in a symmetry-imposed energetic barrier.^1,2^

**Scheme 1:**
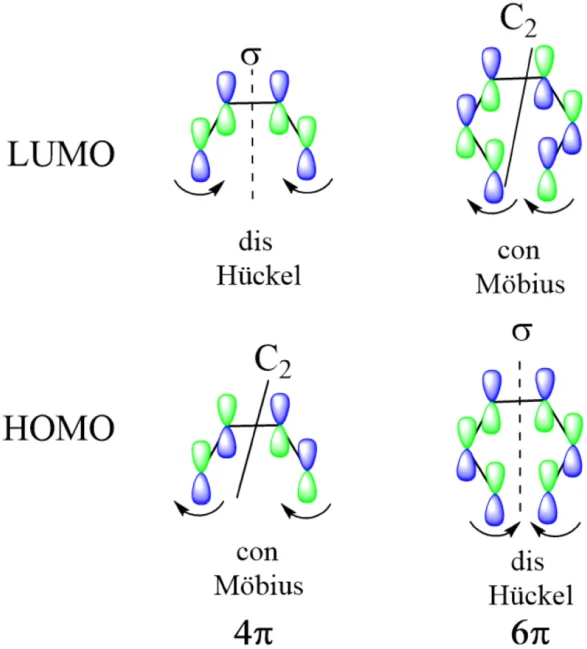
Phase Continuity of Overlapping Orbital Envelopes in the HOMO Requires Conrotatory (Möbius Topology) and Disrotatory (Hückel Topology) Motion in 4π-Electron and 6π-Electron Systems, Respectively[Fn sch1-fn1]

i Electronic excitation to the LUMO reverses phase relationships and inverts the sense of orbital rotations. A 2-fold axis of rotation (*C*_2_) is preserved in a conrotatory motion and a plane of symmetry (σ) is preserved in a disrotatory motion.

Interestingly, soon after the WH paper,^1^ Longuet-Higgins and Abrahamson^3^ presented a complementary approach to analyze the allowedness of electrocyclic reactions, which relied on constructing orbital and state correlation diagrams of species involved in the reaction. Specifically, as shown in Scheme 1, they noted that symmetry elements, such as a 2-fold axis of symmetry (C_2_) or a plane of symmetry (σ), are conserved throughout the reaction, and proposed that the symmetry (*A*/*A*′) or antisymmetry (*B*/*A*″) of the MOs relative to the conserved symmetry element must be maintained. As shown in Scheme 2, such conservation in WH-forbidden reactions requires that the bonding MOs of the reactant correlate with the antibonding MOs of the product, whereas in WH-allowed reactions, the bonding MOs correlate with the bonding MOs. In their landmark paper in 1969,^2^ Woodward and Hoffmann extended these ideas to other classes of concerted pericyclic reactions and formalized the maintenance of bonding continuity as a governing principle in determining the favorability of a reaction pathway.

**Scheme 2:**
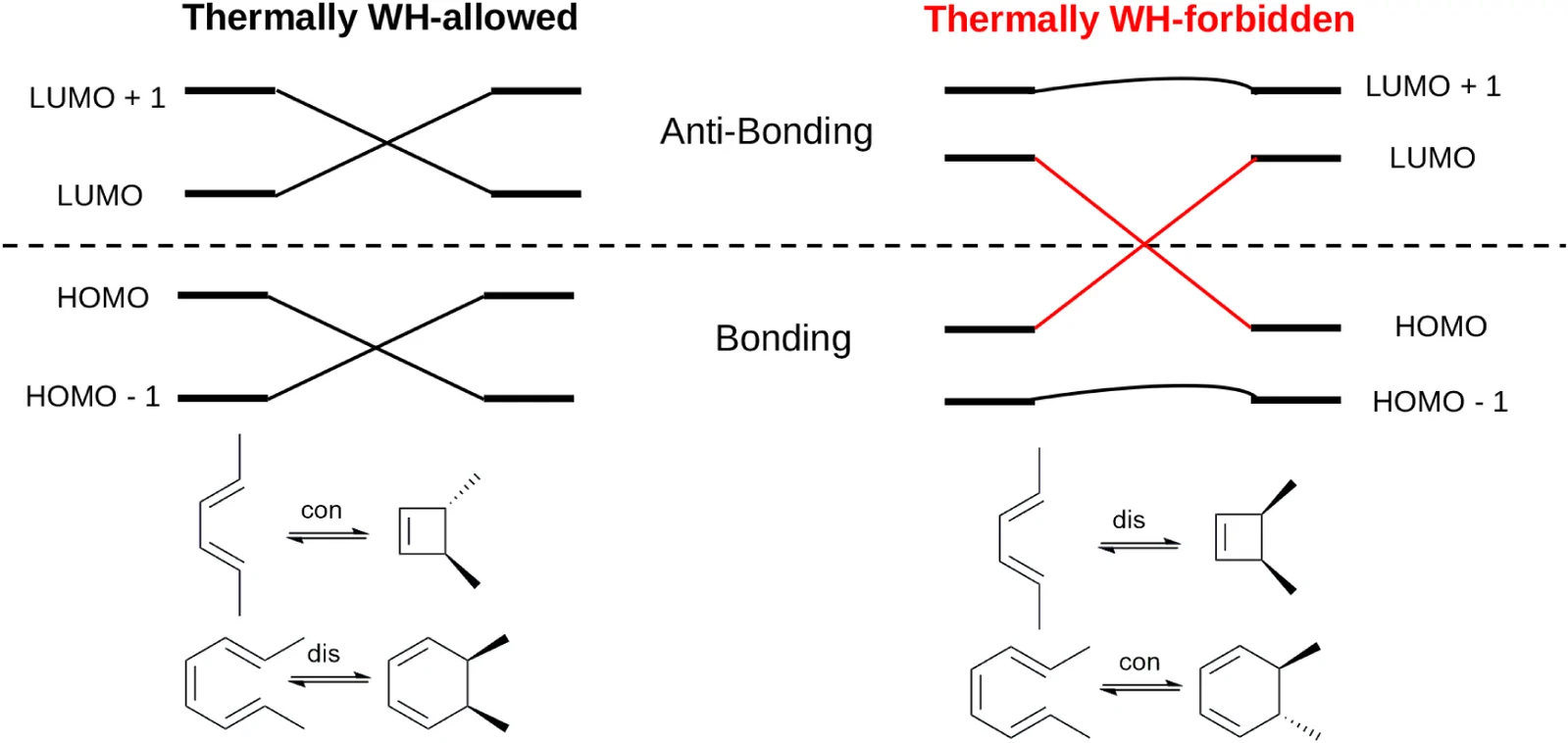
Orbital Correlation Diagrams for Thermally WH-Allowed and WH-Forbidden Reactions in 4π-Electron and 6π-Electron Systems[Fn sch2-fn2]

a Allowed reactions involve correlations of bonding orbitals of reactants with bonding orbitals of products, whereas forbidden reactions involve correlations between bonding orbitals of reactants with antibonding orbitals of products.

A complementary approach to determine the allowedness (or forbiddenness) of a pericyclic reaction was formulated by Dewar and Zimmermann,^4–6^ which involves analyzing the aromatic character of the TS mediating the reaction. They demonstrated that WH-allowed reactions proceed through aromatic transition states (TSs), whereas WH-forbidden reactions involve antiaromatic TSs. The equivalence between Dewar–Zimmermann and Woodward–Hoffmann approaches can be understood in terms of preserving phase continuity in the HOMO. Specifically, phase continuous reaction pathways are mediated by aromatic TSs, while phase discontinuous pathways involve antiaromatic TSs. In the case of (4*n*+2)­π-electron systems, Hückel topology facilitates phase continuity (aromaticity), whereas Möbius topology causes phase discontinuity (see Scheme 1).^5^ For 4*n*π-electron systems, the situation is reversed in that Möbius topology provides aromaticity.

In a recent study by Hoffmann, Seeman, Houk, and coworkers,^7^ by employing multiconfigurational quantum chemical methods to perform intrinsic reaction coordinate (IRC) calculations, they demonstrated that WH-forbidden electrocyclic reactions in 1,3,5-hexatriene and cyclobutene involve crossing between the bonding MOs and antibonding MOs of the open-ring isomer.^7^ This is evident from the significant changes in the occupation numbers of the HOMO and LUMO, with both the orbitals becoming singly occupied along the IRC, reflecting the emergence of an open-shell singlet diradicaloid character. As illustrated in Scheme 2, such crossing implies a disruption in orbital symmetry or bonding continuity, resulting in a kinetically hindered pathway. Such reactions in which the electronic structure changes qualitatively, from a closed-shell singlet form to a broken-symmetry singlet diradicaloid form, have been referred to as “electronic phase transitions″ in the early days of unrestricted Hartree-Fock (UHF) theory literature.^8,9^

Other well-known examples of reactions that involve electronic phase transitions include the dissociation of homodiatomic molecules, such as H_2_, cycloadditions of ethylenes, and *cis*–*trans* isomerizations in olefins.^8,9^ These reactions constitute textbook cases that highlight the inadequacy of the single-configurational, closed-shell singlet description provided by the restricted Hartree-Fock (RHF) wavefunction in electronic structure theory calculations.^8–16^ A well-known remedy for capturing the intermediate regions of the potential energy surface—particularly those involving bond breaking or formation—is to employ a broken-symmetry UHF wavefunction in such regions. Indeed, as extensively documented in the literature,^8–16^ orbital symmetry disruption in electronic phase transitions arising from the mixing of bonding and antibonding MOs, and spin-symmetry breaking associated with the emergence of instability in the RHF solution are equivalent manifestations of the same underlying phenomenon.

Interestingly, recent observations on electrocyclic reactions demonstrated that WH-forbidden TSs, in some cases, possess energies comparable or lower than those of WH-allowed TSs, particularly in highly conjugated hexatrienes^17^ and polarized fulvalenes.^18^ This finding challenges the implicit assumption that forbiddenness necessarily entails a substantial kinetic barrier. In this regard, it is interesting to note that Yamaguchi formulated the orbital-stability rule in the 1980s^9^ which complements the WH rules.^2^ Orbital stability rule states that if the HOMO of the TS is sufficiently stabilized through resonance or electrostatic effects then the reaction becomes kinetically facile owing either to a decrease in diradicaloid character or by following alternative nonradical mechanisms such as zwitterionic pathways.^9,18–22^

In this study, we present a mathematical framework to demonstrate that forbiddenness in electrocyclic reactions can be interpreted as a topological obstruction to bonding disruption in electronic Hilbert space arising from symmetry-constrained unitary evolution of a quantum system. To this end, we first introduce the Unitary Evolution Inaccessibility Theorem (UEIT), which demonstrates that spatial symmetry constraints induce dynamical inaccessibility between two electronic states that belong to orthogonal irreducible representations of the molecular point group. We then generalize this framework to other symmetry constraints commonly employed in electronic structure theory through a broader theoretical formulation, referred to as the Topological Obstruction to Bonding Disruption Theorem (TOBDT). Finally, we demonstrate that a topological obstruction to electronic structure redistribution can be quantified, within the frontier orbital approximation, in terms of a simple measure—the orbital mixing angle Θ—derived through a geometric realization of TOBDT that constitutes a molecular qubit representation of a chemical bond.

## Results and Discussion

### Unitary Evolution Inaccessibility Theorem (UEIT)

**Theorem 1** *Let* H *be a finite-dimensional Hilbert space of many-electron wavefunctions partitioned into two orthogonal subspaces S*_*i*_ *and S*_*j*_, *such that* $H=S_{i}⊕S_{j}$ . *Let Ĥ be a Hermitian, time-independent Hamiltonian of a quantum system that preserves this partitioning. Let* |*ψ*_0_⟩ ∈ *S*_*i*_ *and* |*ψ*_*T*_⟩ ∈ *S*_*j*_ *represent initial and target states of the system, respectively. Then*, |*ψ*_*T*_⟩ *is dynamically inaccessible from* |*ψ*_0_⟩ *under unitary evolution*,

1 Û(t)=exp(−iĤt)

*governed by the Schrödinger equation (adopting atomic units where* ℏ = 1),

2 $$-iĤ\left|\Psi \right\rangle =\frac{\partial \left|\Psi \right\rangle }{\partial t}$$

*In other words, the transition probability from* |*ψ*_0_⟩ *to* |*ψ*_*T*_⟩ *is zero:*

3 $$P_{0\to T}\left(t\right)={|\langle \psi_{T}|Û(t)|\psi_{0}\rangle |}^{2}=0,\forall t>0$$

**Proof** Let the Hilbert space H be partitioned into two orthogonal subspaces *S*_*i*_ and *S*_*j*_ as $H=S_{i}⊕S_{j}$ . Define projection operators $P_{i}$ onto the subspace *S*_*i*_ and $P_{j}$ onto the subspace *S*_*j*_ as

4 $$P_{i}=\underset{i}{\sum }\left|\psi_{i}\right\rangle \left\langle \psi_{i}\right|,\;P_{j}=\underset{j}{\sum }\left|\psi_{j}\right\rangle \left\langle \psi_{j}\right|$$

satisfying $P_{i}$ + $P_{j}$ = *I*, $P_{i}$ $P_{j}$ = 0, $P_{i}^{2}=P_{i}$ , $P_{j}^{2}=P_{j}$ . As the Hamiltonian preserves the Hilbert space partitioning and therefore cannot couple the two subspaces, it commutes with the projection operators $P_{i}$ and $P_{j}$

5 $$\left[Ĥ,P_{i}\right]=0=\left[Ĥ,P_{j}\right]$$

So, it can be expressed as a direct sum of components acting on the individual subspaces, i.e., with *Ĥ*_*i*_ acting only on *S*_*i*_ and *Ĥ*_*j*_ acting only on *S*_*j*_,

6 $$Ĥ=\left[\begin{array}{cc} P_{i}ĤP_{i} & P_{i}ĤP_{j}\\ P_{j}ĤP_{i} & P_{j}ĤP_{j} \end{array}\right]=\left[\begin{array}{cc} {Ĥ}_{i} & 0\\ 0 & {Ĥ}_{j} \end{array}\right]={Ĥ}_{i}⊕{Ĥ}_{j}$$

By expressing the matrix exponential as

7 $$Û\left(t\right)=e^{-i({Ĥ}_{i}⊕{Ĥ}_{j})t}=\overset{\infty }{\underset{n=0}{\sum }}\frac{1}{n!}{\left(-i({Ĥ}_{i}⊕{Ĥ}_{j})t\right)}^{n}=\overset{\infty }{\underset{n=0}{\sum }}\frac{1}{n!}{\left(-i{Ĥ}_{i}t\right)}^{n}⊕\overset{\infty }{\underset{n=0}{\sum }}\frac{1}{n!}{\left(-i{Ĥ}_{j}t\right)}^{n}$$

It follows that unitary evolution under the block-diagonal Hamiltonian preserves the block structure

8 $$Û\left(t\right)=e^{-i{Ĥ}_{i}t}⊕e^{-i{Ĥ}_{j}t}=\left[\begin{array}{cc} e^{-i{Ĥ}_{i}t} & 0\\ 0 & e^{-i{Ĥ}_{j}t} \end{array}\right]$$

For the initial state |ψ_0_⟩ ∈ *S*_*i*_,

9 $$Û\left(t\right)\left|\psi_{0}\right\rangle =\left(e^{-i{Ĥ}_{i}t}⊕e^{-i{Ĥ}_{j}}\right)\left|\psi_{0}\right\rangle =e^{-i{Ĥ}_{i}t}\left|\psi_{0}\right\rangle \in S_{i}$$

As *Û*(*t*) |ψ_0_⟩ ∈ *S*_*i*_ ∀*t*, given any target state |ψ_*T*_⟩ ∈ *S*_*j*_ (*i* ≠ *j*), the transition amplitude integral becomes zero because *S*_*i*_ and *S*_*j*_ are orthogonal subspaces: ⟨ψ_*T*_|*Û*(*t*)|ψ_0_⟩ = 0. This completes the Proof.

In symmetric molecules possessing a point group *G* with a unitary representation ρ:G→U(H) , the Hilbert space H decomposes into group-invariant orthogonal subspaces $H_{\Gamma_{i}}$ corresponding to the irreducible representations (Γ_*i*_) of the point group. Typically, in electrocyclization reactions of symmetric polyenes, a 2-fold axis of rotation *C*_2_ is preserved in a conrotatory process, while a mirror plane σ is preserved in a disrotatory process.^3^ Considering the wavefunctions of the reactant and the TS as basis functions, one can generate the corresponding irreducible representations under the action of the *C*_2_ or *C*_*s*_ symmetry groups.

In the case of formally forbidden reactions, the electronic wavefunction of reactant is a closed-shell singlet, which is symmetric under *C*_2_ (*A*) or *C*_*s*_ (*A*′), while TS is an open-shell singlet diradicaloid, which is antisymmetric under *C*_2_ (*B*) or *C*_*s*_ (*A*″). Because the Hamiltonian operator is invariant under the symmetry operations of the point group, the transition amplitude integral vanishes due to the antisymmetry of the overall integrand, as can be noted from the direct product of the symmetry labels corresponding to irreducible representations of the TS (Γ_TS_), Hamiltonian (Γ_Ĥ_) and the reactant (Γ_reactant_):

10 $$\Gamma_{\mathrm{TS}}⊗\Gamma_{Ĥ}⊗\Gamma_{\mathrm{reactant}}=B⊗A⊗A=B$$

Thus, forbidden reactions correspond to transitions between orthogonal symmetry sectors. In the case of allowed reactions, both the reactant and TS wavefunctions are typically closed-shell singlets, thereby corresponding to transitions entirely within the same symmetry sector.

### Topological Obstruction to Bonding Disruption Theorem (TOBDT)

The symmetry-based formulation of UEIT explains forbidden reactions in idealized high-symmetry molecules in terms of a symmetry-preserving Hamiltonian that cannot connect states belonging to *orthogonal* irreducible representations of the molecular point group. However, in molecules lacking symmetry, such direct representation-theoretic selection rules are no longer operative. This limitation strongly implies the presence of a more fundamental, structurally invariant constraints governing the system. As Woodward and Hoffmann recognized,^2^ the essential invariance is encoded in the bonding continuity of overlapping orbital envelopes, a property that is topological in nature. Therefore, a disruption in bonding continuity along the reaction coordinate constitutes a symmetry-imposed barrier. Below, we formalize and generalize the principle of conservation of orbital symmetry of Woodward and Hoffmann,^2^ through the TOBDT framework, which, unlike UEIT, does *not* require the existence of *orthogonal* symmetry sectors for dynamical inaccessibility between the initial and target states under unitary evolution of a quantum system. In this regard, it is worth noting that other types of symmetry constraints, such as the RHF spin-symmetry constraint discussed in the Introduction, do not necessarily induce a decomposition of Hilbert space into group-invariant orthogonal subspaces. For example, consider a molecular system with two possible frontier orbital configurations: a closed-shell singlet |Ψ_1_⟩ = π^2^π*^0^ and a doubly excited singlet state |Ψ_2_⟩ = π^0^π*^2^. A spin-symmetry-preserving state may be represented as

11 $$\left|\Psi_{+}\right\rangle =\left|\Psi_{1}\right\rangle$$

whereas a symmetry broken state can take the form

12 $$\left|\Psi_{-}\right\rangle =c_{1}\left|\Psi_{1}\right\rangle +c_{2}\left|\Psi_{2}\right\rangle ,\;c_{1},c_{2}\in R,\;c_{1},c_{2}\neq 0$$

As ⟨Ψ_+_|Ψ_–_⟩ = *c*_1_ ≠ 0, these states are not orthogonal. Generalization of UEIT to such constraints therefore requires more involved group-theoretic arguments rooted in unitary matrix groups (Lie groups) and their tangent spaces at identity (Lie algebras), which contain the associated Hamiltonian (scaled by −*i*), the infinitesimal generator of unitary evolution. This makes the Lie-algebraic description essential for understanding quantum dynamics. Readers less acquainted with these mathematical concepts may skip the proof of TOBDT and proceed directly to the chemical interpretation of the proof that follows.

**Theorem2** *Let G* = *U*(*N*) *denote the unitary group acting on the N*-*dimensional Hilbert space* H *of many-electron wavefunctions of a quantum system via the group action:*

13 ·:G×H→H(Ug,|Ψ⟩)→Ug|Ψ⟩

*Suppose* $H_{c}\subseteq H$ *is the reduced manifold that preserves a given symmetry constraint c*. *Let* $S={\mathrm{Stab}}_{G}\left(H_{c}\right)$ *be the setwise stabilizer subgroup of the constraint manifold* $H_{c}$ :

14 $$S={\mathrm{Stab}}_{G}\left(H_{c}\right)=\left\{U_{g}\in G|U_{g}\left(H_{c}\right)=H_{c}\right\}$$

*whose Lie algebra is* s≔Lie(S) . *Suppose* *Ĥ* *is a Hermitian time-independent Hamiltonian of the system. For an arbitrary electronic state* $\left|\Psi \right\rangle \in H_{c}$ , *define the set of all configurations that preserve the symmetry constraint as orbit of* |Ψ⟩

15 $$O_{S}\left|\Psi \right\rangle =\left\{U_{s}\left|\Psi \right\rangle |U_{s}\in S\right\}$$

*and the unconstrained full set of configurations accessible from* |Ψ⟩ *under action of G as*

16 $$O_{G}\left|\Psi \right\rangle =\left\{U_{g}\left|\Psi \right\rangle |U_{g}\in G\right\}$$

*The symmetry-breaking stratum is the set complement obtained by removing the symmetry-preserving set from the full set*

17 $$S_{M}\left|\Psi \right\rangle =O_{G}\left|\Psi \right\rangle \backslash O_{S}\left|\Psi \right\rangle$$

*For an initial state* $\left|\Psi_{0}\right\rangle \in O_{S}\left|\Psi \right\rangle$ *and a target state* $\left|\Psi_{T}\right\rangle \in S_{M}\left|\Psi \right\rangle$ , *no continuous unitary evolution generated by a constraint-preserving Hamiltonian can connect* |Ψ_0_⟩ to |Ψ_*T*_⟩.

***Proof*** Let g=Lie(G) denote the Lie algebra of the full unitary group *G*, and let s=Lie(S) be the Lie algebra associated with the setwise stabilizer subgroup *S* of the constraint-preserving manifold. As demonstrated in the Theoretical Results section of the Supporting Information (SI), it follows from orbit-stabilizer correspondence that the quotient space *G*/*S* can be identified with symmetry-breaking orbit *M*, and we can choose its tangent space **m** to be vector-space complement of **s** yielding

18 g=s⊕m

Note that **m** need not be a Lie subalgebra.

For an arbitrary state |Ψ⟩, the full set of accessible configurations is $O_{G}\left|\Psi \right\rangle$ . However, preserving the symmetry constraint restricts the allowed unitary transformations to those in the subgroup *S*, and the allowed infinitesimal generators to those in **s**. This means the full Hamiltonian (−iĤ∈g) must be projected onto **s** to preserve the constraint. Suppose the projection operators onto subspaces **s** and **m** be $P_{s}$ and $P_{m}$ , and note that they need not commute with the Hamiltonian. Restricting to the constraint-preserving component of the Hamiltonian (*Ĥ*_*s*_), we get

19 $$-i{Ĥ}_{s}\in s$$

Thus, the one-parameter subgroup obtained by exponentiation is contained in *S* (adopting atomic units where ℏ = 1)

20 $$U\left(t\right)=e^{-i{Ĥ}_{s}t}\in S,,\forall t$$

Therefore, for any initial state $\left|\Psi_{0}\right\rangle \in O_{S}\left|\Psi \right\rangle$ , it follows from eq 15 that the time evolved state remains in the symmetry-preserving orbit

21 $$U\left(t\right)\left|\Psi_{0}\right\rangle \in O_{S}\left|\Psi \right\rangle ,\forall t\geq 0$$

For any $\left|\Psi_{T}\right\rangle \in S_{M}\left|\Psi \right\rangle$ , *no* continuous unitary evolution generated solely by a constraint-preserving Hamiltonian can connect |Ψ_0_⟩ to |Ψ_*T*_⟩. This completes the Proof.

It is important to note that the constraint-preserving (*Ĥ*_*s*_) and constraint-breaking (*Ĥ*_*m*_) components of the Hamiltonian do not commute when the interaction term coupling the two components is nonzero, as shown in the Theoretical Results section of the SI. Thus, the total Hamiltonian is not block-diagonal relative to the constraint. As a result, it follows from the Baker–Campbell–Hausdorff formula that the Hamiltonian decomposition is not preserved under exponentiation, which distinguishes TOBDT from UEIT.

#### Chemical Interpretation of the Proof of TOBDT

While TOBDT holds for different types of symmetry constraints as detailed below, bonding disruption in WH-forbidden reactions provides a chemically intuitive interpretation of the mathematical arguments presented in the proof. Within the frontier orbital approximation, the vector-space decomposition g=s⊕m separates infinitesimal generators of unitary transformations into symmetry-preserving generators **s**, which preserve the HOMO double-occupancy constraint (and hence the bonding character), and symmetry-breaking generators **m**, which violate this constraint through HOMO–LUMO mixing. For example, an electronic redistribution that concentrates the HOMO predominantly on one reactive terminus and the LUMO on the other, as in a zwitterionic TS, can occur within the constraint-preserving manifold by retaining the double occupancy of HOMO. Such a wavefunction therefore belongs to the same “orbit″ as the wavefunction of the associated closed-shell reactant or an WH-allowed aromatic TS, and can be reached from it through admissible unitary transformations. In contrast, electronic redistributions involving HOMO–LUMO mixing lie in the symmetry-breaking sector, and therefore remain inaccessible. Such constraint-induced inaccessibility constitutes the topological obstruction to bonding disruption.

The same vector-space decomposition argument can be extended to understand parity selection rules in spectroscopy. For example, in the particle-in-a-box model and in Laporte-forbidden transitions, the parity operator assigns eigenvalues +1 (even) and −1 (odd) to wavefunctions. Unlike the constraint-preserving Hamiltonian operator that entails an even (s:+1) parity, the transition dipole moment operator in spectroscopy has an odd (m:−1) parity. Consequently, transitions between states of the same parity are forbidden, and those between opposite-parity are allowed, as derived in the SI, using the TOBDT framework.

For brevity of discussion, we now classify commonly employed symmetry constraints in electronic structure theory into three distinct types: spatial, spin, and configurational, as described below.

##### Type I: Spin-Symmetry Constraint

The RHF approximation imposes the spin-symmetry condition, i.e., the spatial orbitals for α and β spins must be identical.

22 $$H_{\mathrm{RHF}}=\left\{\left|\Psi \right\rangle :\Phi_{\alpha }=\Phi_{\beta }\right\}$$

Consequently, preserving this constraint requires that unitary transformations on the α and β spins must be identical:

23 $$U_{\alpha }=U_{\beta }$$

The setwise stabilizer subgroup of the constraint-preserving manifold $H_{\mathrm{RHF}}$ ,

24 $$S_{\mathrm{spin}}\left(H_{\mathrm{RHF}}\right)=\left\{U_{g}\in G:U_{g}\left(H_{\mathrm{RHF}}\right)=H_{\mathrm{RHF}}\right\}≅U(N_{\mathrm{occ}}/2{)}_{\mathrm{diag}}\times U(N_{\mathrm{vir}}/2{)}_{\mathrm{diag}}$$

restricts admissible orbital rotations to those preserving spin symmetry. States requiring independent α and β orbital transformations lie outside the RHF orbit. Consequently, broken-symmetry UHF solutions and diradicaloid electronic structures become inaccessible under spin-symmetry-preserving unitary evolution.

##### Type II: Configurational Symmetry Constraint

Within electronic structure theory, the manifold of single Slater determinants is parameterized by the Grassmannian

25 $$Gr\left(N_{\mathrm{occ}},N\right)=U\left(N\right)/\left(U\left(N_{\mathrm{occ}}\right)\times U\left(N_{\mathrm{vir}}\right)\right)$$

whose points correspond to single Slater determinants, with *N*_occ_ and *N*_vir_ being the number of occupied and virtual spin orbitals, respectively (*N* = *N*_occ_ + *N*_vir_). For a given reference determinant |Ψ_0_⟩, such as a closed-shell singlet, the associated configurational symmetry constraint confines the wavefunction to the orbit generated by the pointwise stabilizer subgroup

26 $${Stab}_{G}\left(|\Psi \rangle_{0}\right)=\left\{U_{g}\in G:U_{g}\left|\Psi_{0}\right\rangle =e^{i\chi }\left|\Psi_{0}\right\rangle \right\}≅U\left(N_{\mathrm{occ}}\right)\times U\left(N_{\mathrm{vir}}\right)$$

The stabilizer subgroup consists of independent unitary rotations within the occupied and virtual orbital subspaces, leaving the Slater determinant invariant up to a global phase. Consequently, occupied–virtual mixing is excluded within the configurational symmetry-preserving orbit. Any electronic reorganization requiring occupied–virtual mixing must therefore leave this orbit and move to a different point on the Grassmannian, thereby breaking the configurational symmetry constraint.

##### Type III: Common-Orbital Constraint in Valence Bond Theory

In valence bond theory (VBT), within the valence bond self-consistent field (VBSCF) method, all valence bond (VB) structures are constrained to share a common set of molecular orbitals.^23,24^ The constraint-preserving manifold is

27 $$H_{\mathrm{VBSCF}}=\left\{\left|\Psi \right\rangle \in H|\Phi_{{\mathrm{VB}}_{1}}=\Phi_{{\mathrm{VB}}_{2}}=\cdot \cdot \cdot =\Phi_{{\mathrm{VB}}_{N}}\right\}$$

Consequently, the unitary transformations acting on the orbital space must be identical across all *N* VB structures,

28 $$U_{1}=U_{2}=\cdot \cdot \cdot =U_{N}$$

The setwise stabilizer subgroup of $H_{\mathrm{VBSCF}}$ is the diagonal subgroup

29 $$S_{\mathrm{VBSCF}}\left(H_{\mathrm{VBSCF}}\right)=\left\{U_{g}\in G:U_{g}\left(H_{\mathrm{VBSCF}}\right)=H_{\mathrm{VBSCF}}\right\}≅U\times U\times ...\times U$$

where *U* ∈ *U*(*n*) corresponds to action on a VB structure. The subgroup *S*_VBSCF_ preserves the common-orbital constraint by ensuring that every VB structure undergoes the same unitary transformation and therefore remains within the symmetry-preserving VBSCF orbit. Breaking the common-orbital VBSCF constraint involves action of a larger transformation group as in breathing-orbital valence bond (BOVB) theory

30 $$G_{\mathrm{BOVB}}=\left\{\left(U_{1},U_{2},...,U_{N}\right)|U_{I}\in U\left(n\right),\;I=1,...,N\right\}$$

where each *U*_*I*_ acts independently on the orbital space of the corresponding VB structure, allowing for structure-dependent orbital relaxation.^23,24^ No continuous trajectory generated by the action of *S*_VBSCF_ can reach such states. The trajectory must therefore leave the symmetry-preserving orbit associated with the constraint and enter a symmetry-broken stratum in which each VB structure is allowed to optimize its own orbital representation, thereby recovering the dynamic electron correlation associated with orbital breathing.^23,24^ Within the framework of TOBDT, this suggests that the common-orbital approximation constitutes an imposed constraint whose relaxation alleviates a symmetry-induced topological obstruction to orbital relaxation.

#### Topological Obstructions vs Topological Barriers

Having categorized the distinct spatial, spin, configurational, and common-orbital symmetry constraints within the TOBDT framework, we can now formally distinguish between the geometric notion of a *topological obstruction* and its energetic manifestation as a *topological barrier*. A symmetry-induced topological obstruction exists whenever a symmetry constraint destroys the path-connectedness between an initial and a target state within the constraint-preserving manifold. Equivalently, restricting the admissible unitary-group actions to the stabilizer subgroup associated with the symmetry constraint collapses the continuous family of trajectories in the ambient connected manifold into disconnected loci within the reduced constraint-preserving manifold, rendering the target state inaccessible without symmetry breaking. In electronic structure theory, such an obstruction manifests whenever an electronic redistribution necessarily requires breaking either an intrinsic or an imposed symmetry constraint, such as the spin-symmetry constraint in the RHF wavefunction and common-orbital constraint in the VBSCF wavefunction.

In the case of WH-forbidden electrocyclic reactions, disruption of the bonding continuity of overlapping orbital envelopes necessarily involves breaking both the configurational and spin-symmetry constraints. A topological obstruction is therefore an intrinsic geometric consequence of the underlying symmetry constraint and does not, by itself, imply a large energetic penalty along the reaction coordinate. A topological barrier represents a stronger condition, corresponding to the situation in which a symmetry-induced topological obstruction manifests as a chemically significant kinetic barrier under ambient reaction conditions. This naturally raises the question: under what conditions does a symmetry-induced topological obstruction become a topological barrier? Within the present TOBDT framework, the energetic inaccessibility of the target state is governed by the degree of topological obstruction, which is reflected in the extent of diradicaloid character developed at the TS. It is therefore natural to seek a simple geometric descriptor that quantifies the diradicaloid character and, consequently, the extent of disruption in bonding. A common computational approach for estimating the diradicaloid character (*y*) is employing the Yamaguchi spin-projected UHF (PUHF) formula^25–27^ that uses the UHF natural orbital occupation number of the highest occupied natural orbital (HONO) or the lowest unoccupied natural orbital (LUNO), *n*_H_ and *n*_L_ as

31 $$y=1-\frac{2\left(n_{H}-1\right)}{1+{\left(n_{H}-1\right)}^{2}}$$

32 $$y=1-\frac{2\left(1-n_{L}\right)}{1+{\left(1-n_{L}\right)}^{2}}$$

Interestingly, Sugisaki et al. demonstrated that the diradicaloid character *y* can be transformed into a geometric descriptor Θ that can be employed to prepare Θ-parametrized unitary quantum circuits in quantum computing simulations of singlet diradicaloid states in modeling homolytic dissociation reactions, for example, in H_2_, ethane, ethylene, and acetylene.^27^ Here, we show that this parameter emerges naturally from a molecular qubit representation of a chemical bond within the TOBDT framework.

#### Geometric Realization of TOBDT: A Molecular Qubit Representation

To derive a geometric description of disruption in bonding established by TOBDT, we restrict the electronic structure problem to the frontier-orbital subspace spanned by HONO (|*H*⟩) and LUNO (|*L*⟩). In this subspace, a spin-conserved chemical bond can be mapped onto a two-electron, two-orbital subspace, analogous to the minimal description of bonding in molecular hydrogen. This mapping allows us to represent the chemical bond as a molecular qubit, where the closed-shell ground-state singlet (CS: |{*H* ^2^ *L* ^0^⟩) and the doubly-excited singlet (DX: |{*H* ^0^ *L* ^2^⟩) configurations serve as the computational basis states of the Hilbert space $H≅S^{3}$ . Within this representation, the 3-sphere *S* ^3^ is restricted to the singlet subspace: every state on *S* ^3^ remains a singlet as it is a linear combination of the two singlet states. Since our representation is formulated in terms of spatial orbitals rather than spin orbitals, changes in total spin, such as interconversion between a singlet diradicaloid and a triplet diradicaloid, cannot be accommodated. For brevity of notation, the CS and DX singlet states will hereafter be denoted as |*H*⟩ and |*L*⟩, respectively.

33 $$\left|H^{2}L^{0}\right\rangle \equiv \left|H\right\rangle \equiv \left|0\right\rangle =\left[\begin{array}{c} 1\\ 0 \end{array}\right],,\left|H^{0}L^{2}\right\rangle \equiv \left|L\right\rangle \equiv \left|1\right\rangle =\left[\begin{array}{c} 0\\ 1 \end{array}\right]$$

Any normalized state within H can be expressed as

34 |Ψ⟩=α|H⟩+β|L⟩

where α and β are complex coefficients satisfying |α|^2^ + |β|^2^ = 1. Restricting to real wavefunctions, a normalized state can be expressed either as a symmetric (in-phase) or antisymmetric (out-of-phase) combination of the basis states. By expressing |*H*⟩ = |*H*(1)*H*(2)⟩ and |*L*⟩ = |*L*(1)*L*(2)⟩ in terms of linear combination of atomic orbitals |ϕ_*A*_⟩ and |ϕ_*B*_⟩ centered on atoms *A* and *B*, it can be inferred that the symmetric combination corresponds to an ionic family of wavefunctions |Ψ_ionic_⟩ while an antisymmetric combination yields a covalent family |Ψ_cov_⟩:

35 $$\left|\Psi_{\mathrm{ionic}}\left(\Theta \right)\right\rangle =\cos\Theta \left|H\right\rangle +\sin\Theta \left|L\right\rangle =\cos\Theta \left|\phi_{A}\left(1\right)\phi_{A}\left(2\right)\right\rangle +\sin\Theta \left|\phi_{B}\left(1\right)\phi_{B}\left(2\right)\right\rangle$$

36 $$\left|\Psi_{\mathrm{cov}}\left(\Theta \right)\right\rangle =\cos\Theta \left|H\right\rangle -\sin\Theta \left|L\right\rangle =\cos\Theta \left|\phi_{A}\left(1\right)\phi_{B}\left(2\right)\right\rangle -\sin\Theta \left|\phi_{A}\left(2\right)\phi_{B}\left(1\right)\right\rangle$$

Orbital rotations within the Hilbert space H are parameterized by the mixing angle Θ (see Figure 1) and are realized by unitary 2 × 2 matrices of the *SU*(2) Lie group, whose infinitesimal generators are the Pauli matrices {σ_*x*_, σ_*y*_, σ_*z*_}, scaled by −*i*, forming a basis of the su(2) Lie algebra. Within the spin-symmetry constraint, the su(2) lie algebra decomposes into constraint-preserving (**s**) and constraint-breaking (**m**) components as

37 $$\mathrm{su}\left(2\right)=s⊕m=span\left\{\sigma_{z}\right\}⊕span\left\{\sigma_{x},\sigma_{y}\right\}$$

where σ_*z*_ does not commute with σ_*x*_ or σ_*y*_:

38 $$\left[\sigma_{z},\sigma_{x}\right]=2i\sigma_{y}\neq 0,,\left[\sigma_{y},\sigma_{z}\right]=2i\sigma_{x}\neq 0$$

**Figure 1:**
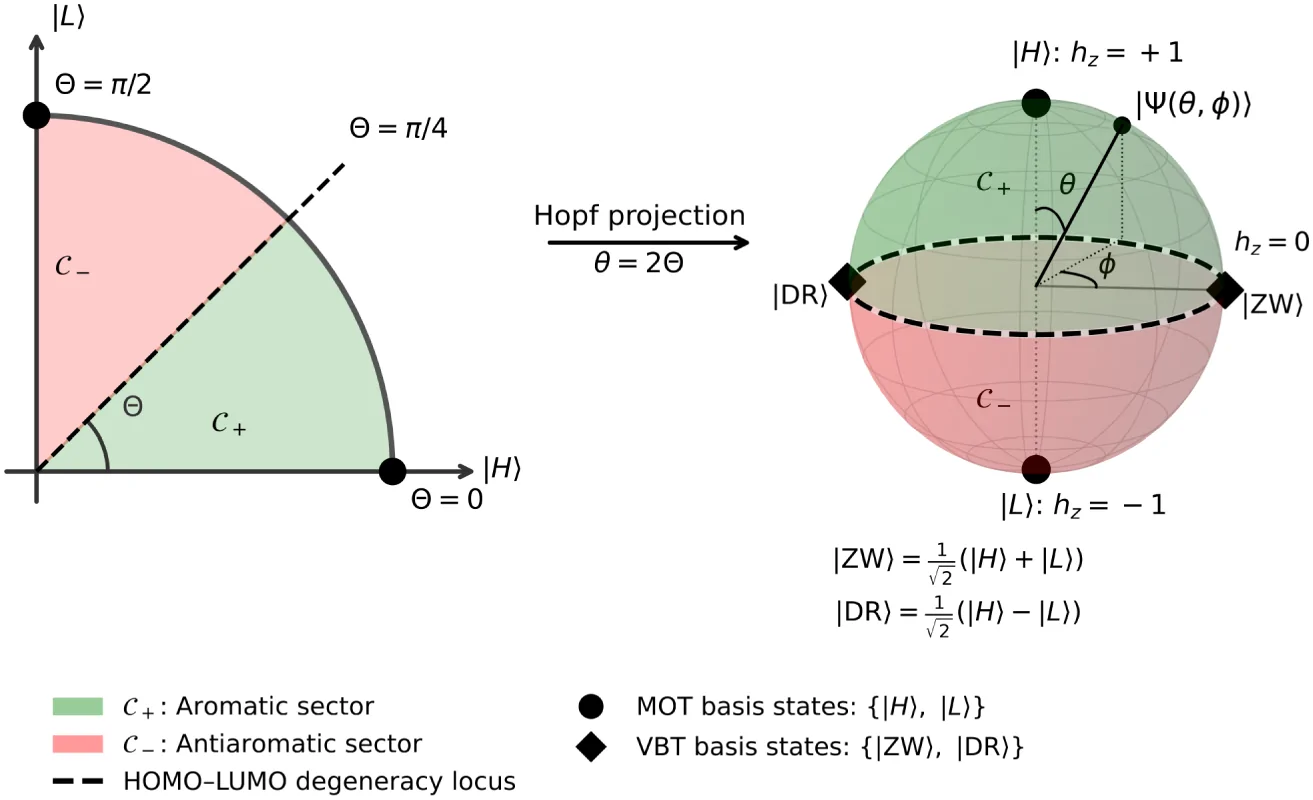
A molecular qubit representation of a chemical bond within the frontier orbital subspace. The left panel shows a representative circular arc (*S* ^1^ ⊂ *S* ^3^) parameterized by the by the Hilbert-space mixing angle Θ ∈ [0, π/2] in the molecular orbital theory (MOT) basis: {|*H*⟩,|*L*⟩}. The right panel shows the resulting Bloch sphere $S^{2}≅{\mathrm{CP}}^{1}$ obtained by the Hopf projection. The poles of the Bloch sphere correspond to the MOT basis: {|*H*⟩,|*L*⟩}, and the equator of the Bloch sphere (θ = π/2, *h_z_* = 0) corresponds to the HOMO–LUMO degeneracy locus, which hosts the zwitterionic and diradicaloid VBT basis states: {|ZW⟩,|DR⟩}. The equator partitions the sphere into aromatic $\left(C_{+}\right)$ and antiaromatic $\left(C_{-}\right)$ sectors. The relation θ = 2Θ connects the parameterizations of the two spaces. Note that aromatic and antiaromatic sector labels shown here correspond to a (4*n*+2)­π-electron system possessing a Hückel topology.

This noncommutativity means that the constraint-preserving generator (σ_*z*_) and the constraint-breaking generators (σ_*x*_, σ_*y*_) cannot be simultaneously diagonalized. As any ${Ĥ}_{s}\in s$ and ${Ĥ}_{m}\in m$ can be expressed as linear combinations of corresponding Pauli basis elements, it follows that Hamiltonian components do not commute and the decomposition *Ĥ* = *Ĥ*_*s*_ + *Ĥ*_*m*_ does not lift to the unitary evolution operator, which explains why projection of *Ĥ* to *Ĥ*_*s*_ is required in the proof of TOBDT.

The natural orbital occupations for the HONO (*n*_H_) and LUNO (*n*_L_) within the *SU*(2) subspace are eigenvalues of the one-electron reduced density matrix

39 $$n_{H}=2\cos^{2}\Theta$$

40 $$n_{L}=2\sin^{2}\Theta$$

This provides an analytic expression for Θ as a smoothly decreasing function of *n*_*H*_ (or a smoothly increasing function of *n*_*L*_), mapping the degree of disruption in bonding onto the *SU*(2) rotation (Θ) of the molecular qubit wavefunction |Ψ_cov_(Θ)⟩ in eq 36. Thus, Θ provides a geometric descriptor of singlet diradicaloid character, wherein the maximal superposition between the CS (Θ = 0 : *n*_H_ = 2, *n*_L_ = 0) and DX ( $\Theta =\frac{\pi }{2}:n_{H}=0$ , *n*_L_ = 2) states corresponds to a pure singlet diradicaloid state with $\Theta =\frac{\pi }{4}$ , and electrons of opposite spins in HONO and LUNO (*n*_H_ = *n*_L_ = 1). The symmetry-induced forbiddenness of an electrocyclic reaction can therefore be defined as the net change in Θ between a TS and the associated reactant

41 $$\Delta \Theta =\Theta_{\mathrm{TS}}-\Theta_{\mathrm{reactant}}$$

A large ΔΘ of around 30° corresponds to a decrease in bonding character of the HONO by ∼25% and a concomitant increase in the antibonding character of LUNO by ∼25%, corresponding to a 50% relative decrease in the formal bond order (cos^2^(ΔΘ) – sin^2^(ΔΘ)). Such large ΔΘ values imply a substantial energetic penalty associated with bonding disruption that characterizes a typical WH-forbidden reaction.

#### Bloch Sphere Representation

As physical states are uniquely defined only up to a global phase, the projective space $S^{2}≅{\mathrm{CP}}^{1}$ can be formally obtained from the Hilbert space via the Hopf fibration^28^ *h*: *S* ^3^ → *S* ^2^ as *S* ^3^/*S* ^1^ ≅ *S* ^2^. The *z*-component of the projection corresponds to the orbital pseudo-spin defined as

42 $$h_{z}=\cos\left(2\Theta \right)=\cos^{2}\Theta -\sin^{2}\Theta =\cos^{2}\frac{\theta }{2}-\sin^{2}\frac{\theta }{2}$$

where θ represents the polar angle on the Bloch sphere *S* ^2^, which is related to the Hilbert space mixing angle Θ by an angle doubling relationship as

43 θ=2Θ

The orbital pseudo-spin *h*_*z*_ also has a direct chemical interpretation as the formal bond order in molecular orbital theory, wherein the CS and DX states map to bond orders of +1 and −1, respectively. On the Bloch sphere, transverse motion away from either the CS (north pole) or DX (south pole) states can be realized through the unitary transformation

44 $$R_{y}\left(\Theta \right)=\exp\left[\Theta \left(-i\sigma_{y}\right)\right]=\exp\left[\Theta \left(a_{L}^{†}a_{H}-a_{H}^{†}a_{L}\right)\right]$$

where σ_*y*_ is the infinitesimal generator of HONO–LUNO mixing, expressed in terms of second-quantized creation (*a* ^†^) and annihilation (*a*) operators. Consequently, the Northern hemisphere (*h*_*z*_ > 0) encompasses wavefunctions that preserve the phase continuity of overlapping orbital envelopes, thereby possessing a predominant bonding character; conversely, the Southern hemisphere (*h*_*z*_ < 0) contains wavefunctions that acquire phase discontinuity or substantial antibonding character. Under the Dewar–Zimmermann theory, these two hemispheres map to aromatic and antiaromatic TS topologies, respectively. Consequently, any continuous reaction pathway connecting electronic states belonging to opposite hemispheres must inevitably intersect the HL-degeneracy equatorial circle *S* ^1^ at which the formal bond order becomes zero (*h*_*z*_ = 0) and the phase becomes undefined.

Interestingly, the HL-degeneracy equatorial circle *S* ^1^ connects the zwitterionic (ZW) and diradicaloid (DR) valence-bond basis states, which within the qubit representation corresponds to Hadamard basis states |+⟩ and |−⟩. This correspondence follows directly by using $\Theta =\frac{\pi }{4}$ in eqs 35 and 36

45 $$\left|\mathrm{ZW}\right\rangle \equiv \left|+\right\rangle =\frac{1}{\sqrt{2}}\left(\left|H\right\rangle +\left|L\right\rangle \right)=\frac{1}{\sqrt{2}}\left[\left|\phi_{A}\left(1\right)\phi_{A}\left(2\right)\right\rangle +\left|\phi_{B}\left(1\right)\phi_{B}\left(2\right)\right\rangle \right]$$

46 $$\left|\mathrm{DR}\right\rangle \equiv \left|-\right\rangle =\frac{1}{\sqrt{2}}\left(\left|H\right\rangle -\left|L\right\rangle \right)=\frac{1}{\sqrt{2}}\left[\left|\phi_{A}\left(1\right)\phi_{B}\left(2\right)\right\rangle +\left|\phi_{B}\left(1\right)\phi_{A}\left(2\right)\right\rangle \right]$$

Within the constraint-preserving manifold $H_{\mathrm{VBSCF}}$ corresponding to the common-orbital VBSCF constraint, the basis states remain orthogonal: ⟨DR|ZW⟩ = 0. The ionic and covalent weights can be evaluated as the squared projections of the molecular qubit wavefunction |Ψ_cov_⟩ on the Hadamard basis states:

47 $$W_{\mathrm{ionic}}=\left\langle \mathrm{ZW}\right|\Psi_{\mathrm{cov}}\rangle^{2}=\frac{1}{2}{[\cos\Theta -\sin\Theta ]}^{2}=\frac{1}{2}\left(1-\sin2\Theta \right)$$

48 $$W_{\mathrm{cov}}=\left\langle \mathrm{DR}\right|\Psi_{\mathrm{cov}}\rangle^{2}=\frac{1}{2}{[\cos\Theta +\sin\Theta ]}^{2}=\frac{1}{2}\left(1+\sin2\Theta \right)$$

For the CS and DX states, these measures correctly yield: *W*_ionic_ = *W*_cov_ = 0.50, consistent with the well-known RHF description of the molecular hydrogen. Within this geometric representation, the Hilbert space mixing angle Θ quantifies the diradicaloid character, and provides a means to estimate covalent and ionic weights through *W*_ionic_ and *W*_cov_ in eqs 47 and 48. This geometric framework will be computationally evaluated by selecting several prototypical examples of WH-allowed and WH-forbidden electrocyclization reactions in the next section.

### Computational Verification of the Molecular Qubit Framework

To computationally verify the molecular qubit framework, we parameterize the wavefunction |Ψ_cov_(Θ)⟩ in terms of Θ derived from the occupation number of the HONO using eq 39, by performing complete active space self-consistent field (CASSCF) calculations employing an active space of 10 electrons in 10 orbitals, as further detailed in the Computational Details section of the SI. For this purpose, four prototype 6π-electrocyclic reactions were chosen to span three mechanistically distinct classes of TSs: antiaromatic TS (AA-TS), aromatic TS (A-TS), and ZW-TS. As illustrated in Scheme 3, **1** and **2** are diarylethenes (DAEs), featuring a cyclohexene and a benzene motif as the bridge between the two aryl units, respectively, which undergo thermal electrocyclization through WH-forbidden AA-TS (conrotatory) or WH-allowed A-TS (disrotatory).^29–31^ Additionally, **1** and **2** are chosen to be isomeric so that the effect of the introduction of the benzene bridge on AA-TS and A-TS can be evaluated. In contrast, the second class constitutes polarized fulvalenes **3**: vinylogous sesquifulvalene and **4**: 6-triafulvenylfulvene, which were selected on the basis of interesting recent observations by Houk, Hoffmann, Seeman, and coworkers.^7,18^ They reported that **3** and **4** prefer conrotatory pathways over the disrotatory pathways, owing to strong polarization of the electronic structure and significant zwitterionic character in the TS. Specifically, they demonstrated that polarization induces changes in the nodal properties of the frontier orbitals by concentrating the HOMO on one of the bond-forming termini, while introducing a node on the other terminus, rendering the orbital phase undefined at the latter. This eliminates the orbital symmetry restrictions of the Woodward–Hoffmann rules and renders the bonding continuity arguments, as well as the aromatic/antiaromatic TS classification inapplicable. These observations are corroborated by our NICS_*zz*_ calculations summarized in Table 1 and the NICS_*zz*_-scan plots presented in Figure S1 of the Supporting Information. Specifically, the TSs of polarized fulvalenes **3** and **4** exhibit only small NICS values, around 8 and 5 ppm for conrotatory TSs, and around −8 and −4 ppm for disrotatory TSs, indicating the absence of significant aromatic or antiaromatic character. In contrast, for **1** and **2**, the conrotatory TSs exhibit large positive NICS values of nearly 31 and 19 ppm, respectively, indicative of significant antiaromaticity, while the disrotatory TSs show large negative NICS values consistent with the expected aromatic character. Additionally, as shown in Table S1 of the Supporting Information, the broken-symmetry UM06-2X wavefunctions for the conrotatory TSs of **1** and **2** exhibit ⟨*S* ^2^⟩ values of 0.7–0.8, together with unpaired spin densities of opposite signs at the bond-forming termini. These results are consistent with the singlet diradicaloid character of these TSs.

**Table 1:** Nucleus Independent Chemical Shift Values at 1.5 Å above the Plane Defined Relative to the Reactive Hexatriene Core (NICS(1.5)*_zz_*), Hilbert Space Mixing Angles Θ, and the Associated Ionic (*W*_ionic_) and Covalent (*W*_cov_) Weights, Changes in Θ Relative to the Reactant (ΔΘ); Free-Energy Barriers for Electrocyclization (Δ*G* ^‡^, kJ mol^–1^) and Electronic Energy Differences (Δ*E*, kJ mol^–1^) between the Conrotatory and Disrotatory Transition States (TSs)^a^

Molecule	TS	NICS(1.5)_*zz*_	Θ^b^	Δ*Θ*	*W*_ionic_	*W*_cov_	Δ*G* ^‡^	Δ*E* ^c^
**1**	**1**-AA-TS	31.3	38.1°	28.3°	0.01	0.99	208.6	–
	**1**-A-TS	–17.4	15.8°	6.1°	0.24	0.76	168.2	–47.9
**2**	**2**-AA-TS	19.2	35.0°	25.1°	0.03	0.97	201.9	–
	**2**-A-TS	–21.7	21.9°	12.0°	0.15	0.85	208.5	+1.2
**3**	**3**-ZW-TS-*anti*	8.4	18.0°	6.5°	0.21	0.79	93.1	–
	**3**-ZW-TS-*syn*	–7.7	14.2°	2.6°	0.26	0.74	141.0	–15.7
**4**	**4**-ZW-TS-*anti*	5.2	13.3°	–0.7°	0.28	0.72	97.9	–
	**4**-ZW-TS-*syn*	–3.9	15.5°	1.4°	0.24	0.76	136.1	19.3

a All geometry optimizations, energies, and NICS calculations performed at the UM06-2X/def2-TZVP level of theory.

b Θ values are derived from the CAS­(10,10) natural orbital occupation numbers of the HONO using eq 39, and the corresponding *W*_cov_ *W*_ionic_ are obtained using eqs 47 and 48.

c For molecules **1** and **2**: Δ*E* = *E*(A-TS) – *E*(AA-TS). For molecules **3** and **4**: Δ*E* = *E*(ZW-TS-*syn*) – *E*(ZW-TS-*anti*).

**Scheme 3:**
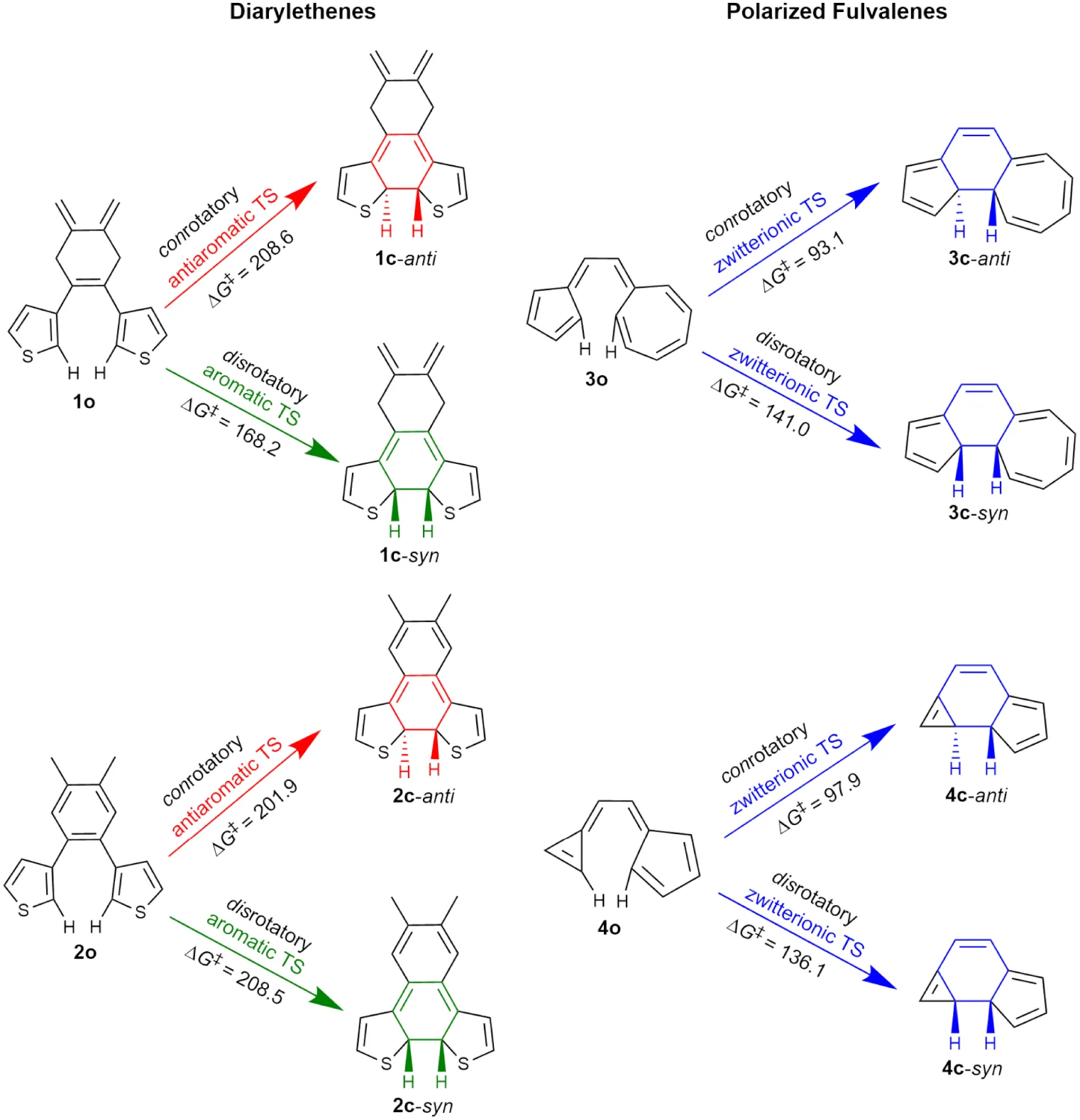
Thermal Electrocyclization Reactions and Associated Free-Energy Barriers (Δ*G* ^‡^, kJ mol^–1^) of **1**–**4** [Fn sch3-fn3]

a (*Left*) Diarylethenes **1** and **2** exhibit conrotatory pathways mediated by WH-forbidden antiaromatic transition states; and disrotatory pathways mediated by WH-allowed aromatic transition states. (*Right*) Polarized fulvalenes **3** and **4** exhibit conrotatory and disrotatory pathways involving zwitterionic transition states (TSs),^7,18^ for which the antiaromatic/aromatic or the forbidden/allowed classification is not applicable.

Using the molecular qubit wavefunction |Ψ_cov_(Θ)⟩, additional insights on stabilizing effects in TSs of **1**–**4** can be obtained by analyzing ionic (*W*_ionic_) and covalent (*W*_cov_) weights (see eqs 47 and 48) listed in Table 1. Interestingly, while the AA-TSs of diarylethenes **1** and **2** possess 97–99% diradicaloid character, the corresponding A-TSs acquire significant ionic character (15–24%). The ionic character of A-TSs of diarylethenes is comparable to the ionic characters of both conrotatory (21–28%) and disrotatory (24–26%) TSs of the polarized fulvalenes **3** and **4**. The large ionic character of TSs in polarized fulvalenes is consistent with the Mulliken charges of opposite signs on the fulvene units reported in Table S1 of the Supporting Information, as well as with the Hirshfeld population analysis reported by Zhou and coworkers.^7^ Furthermore, for **1** and **2**, the large ΔΘ values of 28.3° and 25.1° for AA-TSs are consistent with the corresponding large free-energy barriers for electrocyclization of 208.6 and 201.9 kJ mol^–1^ (see Scheme 3), respectively. In the case of **1**, as can be noted from Table 1, the electronic energy difference (Δ*E*) between the AA-TS and A-TS is −47.9 kJ mol^–1^, indicating that the disrotatory pathway is kinetically favored under ambient conditions. In contrast, replacing the cyclohexene π-linker in **1** with an aromatic benzene π-linker in **2** substantially reduces the energetic separation, yielding a Δ*E* value of only 1.2 kJ mol^–1^ and thereby removing the kinetic distinction between the two pathways.

To investigate whether the introduction of the aromatic benzene bridge exerts differential effects on the energetics of AA-TS and A-TS, isomerization stabilization energies (ISEs) between **1** and **2** were evaluated as illustrated in Scheme S1 of the Supporting Information. Interestingly, the AA-TS exhibits significantly greater stabilization (−175.9 kJ mol^–1^) than the A-TS (−126.8 kJ mol^–1^). These results are consistent with previous observations that AA-TSs exhibit a stronger tendency to be modulated by electron-donating and electron-withdrawing substituents.^17,32^ The preferential stabilization of the AA-TS is also reflected in the smaller Θ of **2** relative to **1** (35.0° vs 38.0°), suggesting that resonance effects stabilize the diradicaloid TS in **2**, in line with our recent observations on diarylbenzenes.^31^ For the polarized fulvalenes **3** and **4**, the situation is markedly different. The corresponding ΔΘ values are only 6.5° and −0.7°, indicating little or no depletion of bonding character at the TS. These results demonstrate that, unlike diarylethenes, conrotatory pathways in polarized fulvalenes do not involve disruption in bonding, owing to strong polarization effects inducing considerable ionic character.

To obtain additional insights into changes in bonding along the reaction pathway, IRC calculations were performed for the conrotatory reaction pathways of all the four systems. Figure 2 presents the corresponding *n*_H_ and *n*_L_ values together with the orbital mixing angle Θ values. For **1** and **2**, Θ increases continuously from around 10° in the reactant to 38.1° and 35.0° at the AA-TS, respectively, consistent with large reduction in the *n*_H_ values and a concomitant increase in the *n*_L_ values, demonstrating progressive disruption in bonding as the TS is approached. In contrast, **3** and **4** exhibit only small variations in both the *n*_H_ and *n*_L_ values, as well as in Θ values. Thus, the IRC profiles reinforce the conclusions drawn from the ΔΘ values in Table 1, namely that substantial depletion of bonding character is a distinguishing feature of the conrotatory pathways of **1** and **2**, whereas no comparable disruption occurs in the corresponding pathways of the polarized fulvalenes **3** and **4**.

**Figure 2:**
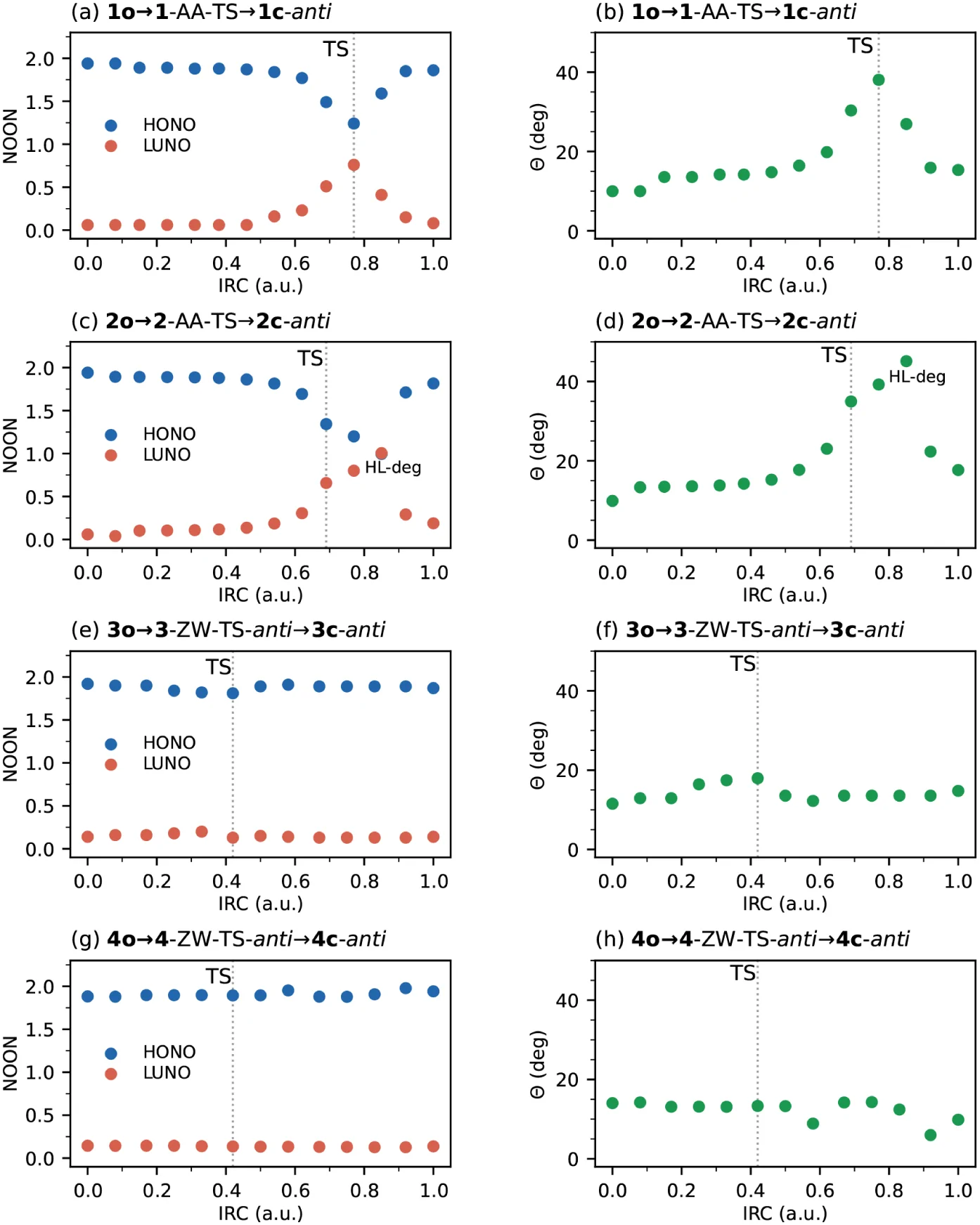
CAS­(10,10) occupation numbers (NOONs) of the highest occupied (HONO, blue) and lowest unoccupied (LUNO, red) natural orbitals (left panel) and the orbital mixing angles Θ (right panel) along the intrinsic reaction coordinate (IRC) of conrotatory electrocyclization pathways. Dotted vertical lines mark the transition state (TS) geometry, and HL-deg indicates HONO-LUNO degeneracy.

Finally, it is of interest to explore whether the free-energy barriers for electrocyclization (shown in Scheme 3) bear correlation with ΔΘ, and if ΔΘ can be obtained in a computationally inexpensive manner. However, as free-energy barriers are likely to be sensitive to conformational factors and electronic factors beyond the bonding disruption, we considered results only for conrotatory pathways of **1**–**4** to facilitate a balanced comparison. As illustrated in Figure S2 of the Supporting Information, the free-energy barriers show a linear correlation with ΔΘ with a correlation coefficient of 0.97, suggesting that ΔΘ serves as a geometric descriptor of the degree of forbiddenness for these reaction pathways. Furthermore, we compared Θ values obtained using different active spaces in CASSCF calculations for AA-TS of **2** in Table S1 of the Supporting Information. These results show good convergence behavior of Θ at the CAS­(10,10) level, with the smaller active spaces underestimating by 3–6°. Alternatively, Θ can be calculated by using the Yamaguchi PUHF formula^25–27^ in eq 32 for diradicaloid character (*y*) and then using the relation

49 $$\Theta =\sin^{-1}\sqrt{\frac{y}{2}}$$

Interestingly, as can be noted from Table S2 of the Supporting Information, Θ values obtained from eq 49 provide good agreement (within 3–4°) with corresponding CAS­(10,10) values for AA-TSs of **1** and **2**. Overall, it can be concluded that the orbital mixing angle Θ and the molecular qubit wavefunction |Ψ_cov_(Θ)⟩ provide a computationally accessible geometric realization of the TOBDT framework, quantifying symmetry-induced topological obstructions to bonding disruption in electrocyclization reactions and distinguishing diradicaloid from zwitterionic pathways.

## Conclusions

In this work, we have generalized the well-known Woodward–Hoffmann rules to different types of symmetry constraints by proposing two complementary group-theoretic formulations: UEIT and TOBDT. In molecules possessing spatial symmetry, UEIT shows that when the initial and target states belong to distinct irreducible representations (Γ_*i*_) of the molecular point group, the Hamiltonian becomes block-diagonal and unitary evolution cannot transfer amplitude between group-invariant orthogonal subspaces. TOBDT generalizes this principle beyond point-group symmetry by demonstrating that a topological obstruction to bonding disruption constitutes a trajectory emanating from a symmetry-preserving orbit being forced to leave the orbit to reach a target state residing in the symmetry-breaking stratum. Confining to the frontier orbital subspace, through a molecular qubit description of chemical bond, we have demonstrated that such motion in WH-forbidden electrocyclization reactions involves traversing the surface of the Bloch sphere away from the poles, corresponding to changes in the orbital mixing angle Θ, which quantifies the extent of bonding disruption. As a result, large ΔΘ values impose a considerable energetic penalty.

The molecular qubit framework of TOBDT is validated through calculations on four prototype electrocyclization reactions spanning three mechanistically distinct classes of WH-forbidden TSs: AA-TSs, where Θ increases substantially along the reaction coordinate; A-TSs and ZW-TSs, where changes in Θ remain small, correctly identifying the absence of bonding disruption along the reaction coordinate. Thus, the TOBDT framework transforms the well-known Woodward–Hoffmann rules into a quantitative geometric parameterization Θ, thereby facilitating an improved electronic description of WH-forbidden TSs through a simple two-configurational wavefunction rooted in the PUHF formalism.^25^ Finally, the molecular qubit representation of TOBDT naturally provides chemically-motivated trial states for quantum phase estimation in quantum computing,^27^ prepared from a closed-shell reference through the unitary transformation *R*_*y*_(Θ) = exp­[Θ­(−*i*σ_*y*_)], where σ_*y*_ is the Pauli matrix that serves as the infinitesimal generator of HOMO–LUMO mixing.

## Supplementary Material



## Data Availability

The data underlying this study are available in the published article and its Supporting Information.

## Notes

Cartesian coordinates of optimized geometries, Lie algebra decomposition and Hamiltonian projection in TOBDT, $Z_{2}$ -graded symmetric decomposition and parity selection rules, ISE scheme, NICS_*zz*_-scan, Δ*G* ^‡^ vs Θ plot, spin densities and Mulliken charges, Θ-convergence, and computational details (PDF)
