# Supplementary material for: Symmetry-Induced Topological Obstructions in Woodward–Hoffmann Forbidden Reactions: A Molecular Qubit Representation
Source: J Phys Chem A. 2026 Sep 15;130(38):7732–43. doi: 10.1021/acs.jpca.6c04534 (PMC13618050; doi:10.1021/acs.jpca.6c04534)
Supplement: Supplementary file 1 [file jp6c04534_si_001.pdf]

Symmetry-Induced Topological Obstructions in Woodward–Hoffmann Forbidden Reactions: A Molecular Qubit Representation

Subha Sandeep Repaka,[†] Thillaiarasi Sukumar,[‡] Bhagyashri Devaru Bhat,[¶] Ran Friedman,[*,§] and Baswanth Oruganti[*,‡,‖]

†*Department of Foundational Sciences (Mathematics), Ramaiah Institute of Science and Management, Bengaluru 560064, India.*

‡*Department of Chemistry, SRM University-AP, Amaravati 522240, India.*

¶*School of Mathematics and Natural Sciences, Chanakya University, Bengaluru 562165, India.*

§*Department of Chemistry and Biomedical Sciences, Linnaeus University, SE-391 82 Kalmar, Sweden.*

‖*Department of Mathematics and Mathematical Statistics, Umeå University, SE-901 87 Umeå, Sweden.*

E-mail: ran.friedman@lnu.se; baswanth.oruganti@gmail.com

Contents

Computational Details **S3**

DFT Geometry Optimizations and Frequency Calculations S3

DFT NICS Calculations S4

DFT IRC Calculations S4

CASSCF Calculations S5

Convergence of Orbital Mixing Angle Θ S5

Theoretical Results **S6**

Lie Algebra Decomposition and Hamiltonian Projection in TOBDT S6

$\mathbb{Z}_2$-Graded Symmetric Decomposition and Parity Selection Rules S8

Scheme S1 **S12**

Table S1 **S13**

Table S2 **S14**

Figure S1 **S15**

Figure S2 **S16**

References **S17**

Cartesian Coordinates **S18**

Computational Details

DFT Geometry Optimizations and Frequency Calculations

The electrocyclization reactions of **1**–**4** were modeled by performing DFT calculations as follows. Geometry optimizations of the open-ring (**o**) and closed-ring (**c**:*anti* and *syn*) forms were carried out using the M06-2X functional[1] and the def2-TZVP basis set. Transition structures (TSs) corresponding to the conrotatory and disrotatory reaction pathways were optimized using the broken-symmetry unrestricted M06-2X (UM06-2X) functional with the def2-TZVP basis set. For the disrotatory pathways of **1** and **2**, and for both conrotatory and disrotatory TSs of **3** and **4**, the unrestricted solution collapsed to the corresponding symmetry-preserving restricted solution, indicating the absence of symmetry breaking and confirming the closed-shell character of these TSs. Starting from the optimized geometries, vibrational frequency calculations were performed at the same level of theory to confirm the nature of all stationary points. The open-ring and closed-ring (*anti* and *syn*) forms were verified to be local minima by the absence of any imaginary vibrational frequency. Each TS exhibited a single imaginary frequency corresponding to the motion along the reaction coordinate.

The electronic energy differences between the antiaromatic-TS (AA-TS) and aromatic-TS (A-TS) for **1** and **2** were computed as

$$\Delta E = E(\text{A-TS}) - E(\text{AA-TS}). \tag{1}$$

Similarly, the electronic energy differences between the zwitterionic ZW-TS-*anti* and ZW-TS-*syn* for **3** and **4** were computed as

$$\Delta E = E(\text{ZW-TS-syn}) - E(\text{ZW-TS-}anti). \tag{2}$$

To quantify how introduction of an aromatic π-linker influences the energetics of the AA-

TS and A-TS, **1** is transformed into its isomeric form **2** by replacing the non-aromatic cyclohexene π-linker with an aromatic benzene π-linker. The differential electronic effects on the A-TS and AA-TS are quantified by isomerization stabilization energies (ISEs) defined as

$$\mathrm{ISE_{AA}} = E_{\mathrm{AA}}(\mathbf{2}) - E_{\mathrm{AA}}(\mathbf{1}), \tag{3}$$

$$\mathrm{ISE_{A}} = E_{\mathrm{A}}(\mathbf{2}) - E_{\mathrm{A}}(\mathbf{1}). \tag{4}$$

DFT NICS Calculations

Using the DFT optimized TS geometries, nucleus-independent chemical shift (NICS) calculations were performed at the UM06-2X/def2-TZVP level using the gauge-including atomic orbitals (GIAO) formalism, and using a broken-symmetry guess wavefunction. The NICS_{zz} component along the normal to the plane containing reactive hexatriene core was evaluated by placing ghost atoms at distances of 0.00, 1.00, 1.25, 1.50, 1.75, and 2.00 Å from the ring critical point (RCP). The reference plane was defined using the RCP and the two central carbon atoms of the bridge connecting the two ring systems. The RCP was located using atoms-in-molecules (AIM) analysis[2] implemented in the Multiwfn software package[3] applied to the UM06-2X/def2-TZVP wavefunction. The NICS_{zz} component was extracted from the full shielding tensor σ as

$$\mathrm{NICS}_{zz} = -\langle n|\, \sigma \,|n\rangle\,, \tag{5}$$

where $|n\rangle$ is the unit normal vector to the reference plane.

DFT IRC Calculations

Intrinsic reaction coordinate (IRC) calculations were performed for the conrotatory reaction pathways of **1**–**4** at the UM06-2X/def2-TZVP level of theory. The IRC was initiated from

each conrotatory TS using the Hessian computed at the TS geometry and propagated both in the forward and reverse directions using the least quadratic approximation and with a step size of 0.10 Bohr for a maximum of 100 steps, with the `Guess=Mix` keyword applied throughout to maintain the broken-symmetry unrestricted description along the reaction coordinate. From the resulting geometries, a few representative geometries distributed uniformly over the reaction coordinate are chosen for further analysis using complete active space self-consistent field (CASSCF) calculations.

All DFT calculations were performed using the GAUSSIAN 16 program.[4]

CASSCF Calculations

Using DFT optimized geometries, CASSCF[5,6] singlepoint calculations were performed using the OpenMolcas software package[7] with the def2-SVP basis set. Specifically, for the IRC geometries, CASSCF singlepoint calculations were performed on the representative geometries along the conrotatory IRC of molecules **1**–**4**, and only on the open-ring form and TS in the case of the disrotatory pathways. The active space comprised ten π electrons in ten π orbitals, CAS(10,10). The natural orbital occupation number (NOON) of the highest occupied natural orbital (HONO) obtained from the CASSCF density matrix were used to compute the orbital mixing angle Θ, the covalent weight W_{cov}, and the ionic weight W_{ion} as described in the main text.

Convergence of Orbital Mixing Angle Θ

To assess the convergence of Θ with respect to active space size, a series of CASSCF/def2-SVP single-point calculations with active spaces ranging from CAS(2,2) to CAS(12,12) were performed on the UM06-2X/def2-TZVP optimized geometry of the AA-TS of **2** using the PySCF program.[8] Additionally, unrestricted Hartree–Fock (UHF) singlepoint calculations were performed on the AA-TSs of molecules **1** and **2** using Gaussian 16 to extract Θ using the Yamaguchi spin-projection formula described in the main text.

Theoretical Results

Lie Algebra Decomposition and Hamiltonian Projection in TOBDT

Let $G = U(N)$ act on the finite-dimensional Hilbert space $\mathcal{H}$ of many-electron wavefunctions. Let $\mathcal{H}_c \subset \mathcal{H}$ denote the constraint-preserving manifold associated with a given symmetry constraint c. We define the setwise stabilizer subgroup of the constraint manifold as

$$S = \mathrm{Stab}_G(\mathcal{H}_c), \tag{6}$$

with Lie algebra

$$\mathfrak{s} = \mathrm{Lie}(S) \subseteq \mathfrak{g} = \mathfrak{u}(N). \tag{7}$$

The tangent space of a smooth manifold at a point is the vector space of tangent vectors to the smooth curves passing through that point. The tangent space of G at the identity $e = I$ is

$$T_I G = \{\gamma'(0) \,|\, \gamma : (a, b) \to G,\ \gamma(0) = I\}, \tag{8}$$

which is the Lie algebra $\mathfrak{g}$. Quotienting the group G with stabilizer subgroup S, we obtain the quotient space G/S, which parametrizes directions in G transverse to the constraint-preserving subgroup S. Note that since S may not be a normal subgroup of G, G/S is a homogeneous space rather than a Lie group. According to orbit-stabilizer correspondence Theorem, the quotient space G/S can be identified with a constraint-breaking orbit, say M. At the identity coset eS, the tangent space of M is

$$T_{eS}(M) = T_{eS}(G/S) \cong \mathfrak{g}/\mathfrak{s}. \tag{9}$$

We therefore identify this tangent space with a vector space complement $\mathfrak{m}$ of $\mathfrak{s}$ $\mathfrak{m} \cong \mathfrak{g}/\mathfrak{s}$, giving

$$\mathfrak{g} = \mathfrak{s} \oplus \mathfrak{m}. \tag{10}$$

Here, $\mathfrak{s}$ consists of infinitesimal generators that preserve the constraint, whereas $\mathfrak{m}$ represents infinitesimal generators that break the constraint. This vector space decomposition is the foundation of the TOBDT proof in the main text, where the Hamiltonian is projected onto $\mathfrak{s}$ to obtain the constraint-preserving dynamics. Also, note that the Lie subalgebra $\mathfrak{s}$ is closed under the Lie bracket:

$$[\mathfrak{s}, \mathfrak{s}] \subseteq \mathfrak{s}. \tag{11}$$

Hamiltonian Projection

Define the projection maps onto constraint-preserving $\mathfrak{s}$ and constraint-breaking $\mathfrak{m}$ subspaces of Lie algebra $\mathfrak{g}$

$$\mathcal{P}_{\mathfrak{s}} : \mathfrak{g} \to \mathfrak{s}, \tag{12}$$

$$\mathcal{P}_{\mathfrak{m}} : \mathfrak{g} \to \mathfrak{m}, \tag{13}$$

such that for any $\hat{X} \in \mathfrak{g}$,

$$\hat{X} = \mathcal{P}_{\mathfrak{s}}(\hat{X}) + \mathcal{P}_{\mathfrak{m}}(\hat{X}) = \hat{X}_{\mathfrak{s}} + \hat{X}_{\mathfrak{m}}, \quad \text{where} \quad \hat{X}_{\mathfrak{s}} \in \mathfrak{s}, \quad \hat{X}_{\mathfrak{m}} \in \mathfrak{m}. \tag{14}$$

Thus, the Hamiltonian can be decomposed into constraint-preserving and constraint-breaking components

$$\hat{H} = \hat{H}_s + \hat{H}_m. \tag{15}$$

In general, the constraint-breaking subspace $\mathfrak{m}$ need not be a Lie subalgebra of $\mathfrak{g}$. Furthemore, the commutators $[\mathfrak{m}, \mathfrak{m}]$ and $[\mathfrak{s}, \mathfrak{m}]$ are not required to vanish and can yield components spanning both subspaces.

As an illustrative example in electronic structure theory, consider a two-state Hamiltonian expressed in the basis of the closed-shell singlet (CS) and doubly excited singlet (DX) states, such as in the molecular qubit representation of TOBDT discussed in the main text (or in

the Hartree–Fock stability Hessian). Here, the constraint-preserving ($\hat{H}_s$) and constraint-breaking ($\hat{H}_m$) components of the Hamiltonian take block-diagonal and off-block-diagonal forms, and do not commute (unless V=0).

$$\hat{H}_s = \begin{bmatrix} E_{\text{CS}} & 0 \\ 0 & E_{\text{DX}} \end{bmatrix}, \tag{16}$$

$$\hat{H}_m = \begin{bmatrix} 0 & V \\ V^\dagger & 0 \end{bmatrix}, \tag{17}$$

$$[\hat{H}_s, \hat{H}_m] \neq 0, \tag{18}$$

where V represents interaction energy between the CS and DX states. In such cases, the decomposition $\hat{H} = \hat{H}_s + \hat{H}_m$ is not preserved under exponentiation. This non-lifting behavior distinguishes TOBDT from UEIT, where the Hamiltonian is strictly block-diagonal and the unitary evolution operator inherits the same block structure.

Situations of particular interest in spectroscopy involve probing under what conditions both the commutators $[\mathfrak{m}, \mathfrak{m}]$ and $[\mathfrak{s}, \mathfrak{m}]$ close with a $\mathbb{Z}_2$-graded symmetric decomposition

$$[\mathfrak{s}, \mathfrak{s}] \subseteq \mathfrak{s}, \tag{19}$$

$$[\mathfrak{s}, \mathfrak{m}] \subseteq \mathfrak{m}, \tag{20}$$

$$[\mathfrak{m}, \mathfrak{m}] \subseteq \mathfrak{s}. \tag{21}$$

$\mathbb{Z}_2$-Graded Symmetric Decomposition and Parity Selection Rules

The space of all linear operators on $\mathcal{H}$, denoted $\text{End}(\mathcal{H})$, forms a linear vector space. Its skew-Hermitian elements constitute the generator space of physical observables (scaled by $-i$):

$$\mathfrak{g}(\mathcal{H}) = \{X \in \text{End}(\mathcal{H}) : X^\dagger = -X\}, \tag{22}$$

which is a real Lie algebra under the commutator. Besides the generator space, the set of all normalized electronic wavefunctions spanning a unit hypersphere $S^{2N-1} \subset \mathcal{H}^N$ can be mapped onto this Lie algebra using a corresponding density operator by $\phi : S^{2N-1} \to \mathfrak{g}$ as

$$\phi(|\Psi\rangle) = X_\Psi = -i\,|\Psi\rangle\,\langle\Psi|\,. \tag{23}$$

The image of this map defines the state-operator manifold, $\mathcal{D}(\mathcal{H}) = \phi(S^{2N-1})$, a non-linear sub-manifold embedded within the Lie algebra $\mathfrak{g}(\mathcal{H})$

$$\mathcal{D}(\mathcal{H}) = \{X_\Psi = -i\,|\Psi\rangle\,\langle\Psi| : |\Psi\rangle \in S^{2N-1}\} \tag{24}$$

This embedding maps both observables and states into the same Lie algebra, enabling tracking of how symmetry constraints act on generators and state-operators.

The unitary group $G = U(\mathcal{H})$ acts on $\mathfrak{g}(\mathcal{H})$ via two distinct operations. The first is the adjoint action on the generator space:

$$\mathrm{Ad}_g(X) = gXg^{-1}, \qquad g \in G, \quad X \in \mathfrak{g}(\mathcal{H}), \tag{25}$$

which is a Lie algebra automorphism. The second is the left action on the state-operator manifold:

$$L_g(X_\Psi) = gX_\Psi = -i(g\,|\Psi\rangle)\,\langle\Psi|\,, \qquad g \in G, \quad X_\Psi \in \mathcal{D}(\mathcal{H}). \tag{26}$$

We now consider the case where the symmetry operation is the parity operator U_p, which is a unitary involution satisfying $U_p^\dagger = U_p$ and $U_p^2 = I$. In this case, the pointwise stabilizer subgroup of Lie subalgebra $\mathfrak{s}$ is the group of all involutive unitary transformations that satisfy

$$S = \{U_g \in G : \mathrm{Ad}_g(X) = X; \quad L_g(X_\Psi) = X_\Psi, \quad \forall X \in \mathfrak{s}, \quad \forall X_\Psi \in \mathcal{D}(\mathcal{H} \cap \mathfrak{s})\}. \tag{27}$$

Thus, the stabilizer subgroup S is the centralizer of the Lie subalgebra $\mathfrak{s}$ within the unitary group G.

The adjoint action of U_p on $\mathfrak{u}(\mathcal{H})$ represents an involutive automorphism, with $+1$ and -1 corresponding to constraint-preserving ($\mathfrak{s}$) and constraint-breaking ($\mathfrak{m}$) eigenspaces

$$\mathfrak{g} = \mathfrak{s} \oplus \mathfrak{m}, \tag{28}$$

where

$$\mathfrak{s} = \{X \in \mathfrak{u}(\mathcal{H}) : \mathrm{Ad}_{U_p}(X) = +X\}, \tag{29}$$

$$\mathfrak{m} = \{X \in \mathfrak{u}(\mathcal{H}) : \mathrm{Ad}_{U_p}(X) = -X\}. \tag{30}$$

Because the adjoint action preserves the Lie bracket structure, the commutators satisfy the $\mathbb{Z}_2$-graded symmetric space relations given in Eqs. 19–21. These relations algebraically encode the operator parity selection rules: *the commutator of two even operators is even, of an even and an odd operator is odd, and of two odd operators is even.*

To classify states rather than operators, we utilize the left action of U_p on the state-operator manifold $\mathcal{D}(\mathcal{H})$. For a state operator corresponding to a wavefunction of definite parity, we get

$$L_{U_p}(X_\Psi) = U_p X_\Psi = \pm X_\Psi. \tag{31}$$

Combining parities from both types of actions, we can derive overall parity of the transition dipole moment operator $\hat{T}_{fi} = X_{\Psi_f} \hat{\mu} X_{\Psi_i}$. Acting with U_p scales the individual components by their respective parities:

$$U_p \hat{T}_{fi} = \left(U_p X_{\Psi_f}\right)\left(U_p \hat{\mu} U_p^{-1}\right)\left(U_p X_{\Psi_i}\right). \tag{32}$$

The dipole operator is constraint-breaking ($\hat{\mu} \in \mathfrak{m}$), yielding a parity of -1. Assume that the initial and final electronic states transform with parities p_i and p_f. Taking the direct

product of these component parities, we get

$$p_{\text{total}} = p_f \cdot (-1) \cdot p_i = -p_i p_f. \tag{33}$$

If the initial and final state-operators possess identical parity ($p_i = p_f$), the direct product of parities is odd ($p_{\text{total}} = -1$), rendering the transition forbidden. Conversely, if they possess opposite parities ($p_i \neq p_f$), the direct product of parities is even ($p_{\text{total}} = +1$), and the transition is allowed.

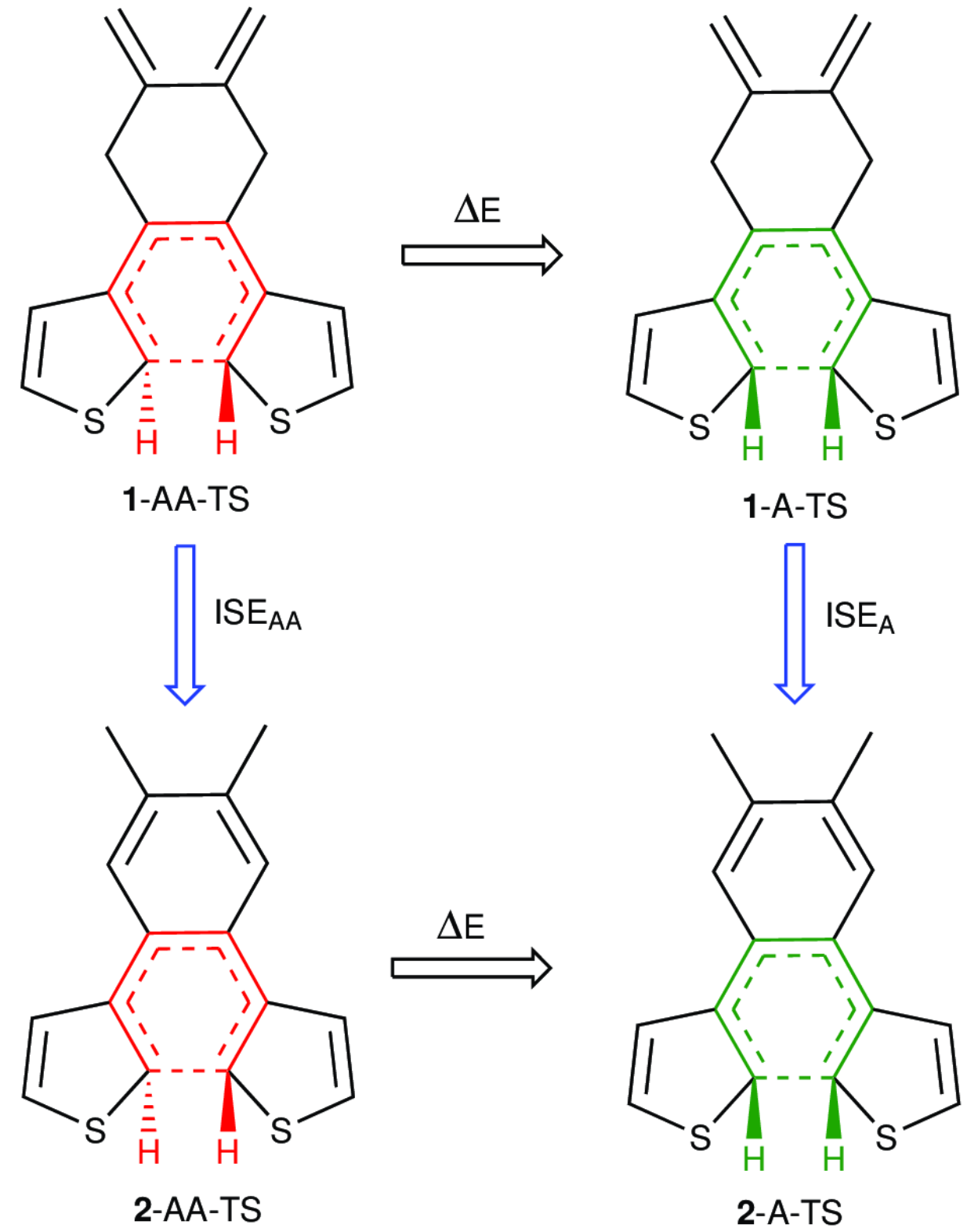

Scheme S1: Structures of the antiaromatic (AA) and aromatic (A) transition states (TSs) of **1** and **2**. Electronic energy difference between AA-TS and A-TS (ΔE) quantifies the stability of the A-TS relative to the AA-TS. Isomerization stabilization energies (ISEs) measure how introduction of an aromatic π-linker influences energetics of each of the TSs.

Table S1: Atomic spin densities on the bond forming termini, expectation values of the spin-squared operator $\langle S^2 \rangle$, and sums of Mulliken charges over heavy atoms on the each thiophene or fulvene unit.[a]

Molecule	Spin densities[b]		$\langle S^2 \rangle$	Mulliken charges[b]	
	C1	C2		Ring A	Ring B
1ts-AA	0.18	−0.18	0.82	−0.02	−0.02
1ts-A	0.00	0.00	0.00	−0.06	−0.05
2ts-AA	0.14	−0.14	0.72	−0.04	−0.04
2ts-A	0.00	0.00	0.00	−0.04	−0.04
3ts-anti	0.00	0.00	0.00	−0.20	0.43
3ts-syn	0.00	0.00	0.00	−0.22	0.34
4ts-anti	0.00	0.00	0.00	0.31	−0.26
4ts-syn	0.00	0.00	0.00	0.29	−0.26

[a] All calculations were carried out at the UM06-2X/def2-TZVP level of theory.

[b] C1 and C2, and ring A and ring B correspond to the left and right thiophene units (in **1** and **2**), and left and right fulvene units (in **3** and **4**), respectively, as shown in Scheme 3 of the main article.

Table S2: CASSCF, UHF and spin-projected UHF (PUHF) natural orbital occupation numbers for the HONO (n_{H}) and LUNO (n_{L}) for the antiaromatic TSs (AA-TSs) of **1** and **2**.

Species	Level of Theory[a]	n_{H}	n_{L}	Θ
**2**-AA-TS	CAS(2,2)	1.53	0.47	29.1°
	CAS(4,4)	1.54	0.46	28.6°
	CAS(6,6)	1.52	0.49	29.5°
	CAS(8,8)	1.44	0.57	32.1°
	CAS(10,10)	1.34	0.66	35.0°
	CAS(12,12)	1.35	0.65	34.7°
	UHF	1.11	0.89	41.9°
	PUHF[b]	1.21	0.79	38.8°
**1**-AA-TS	CAS(10,10)	1.24	0.76	38.0°
	UHF	1.07	0.93	43.0°
	PUHF[b]	1.14	0.86	41.1°

[a] All calculations were performed using the def2-TZVP basis set.

[b] For PUHF, the diradicaloid character y is obtained from the Yamaguchi spin-projection formula

$$y = 1 - \frac{2(n_{\mathrm{H}} - 1)}{[1 + (n_{\mathrm{H}} - 1)^2]},$$

where n_{H} is the UHF natural orbital occupation number of the HONO. The PUHF n_{H} and n_{L} values were subsequently computed as $2 - y$ and y, respectively, with Θ obtained as $\Theta = \sin^{-1}\sqrt{\frac{y}{2}}$.

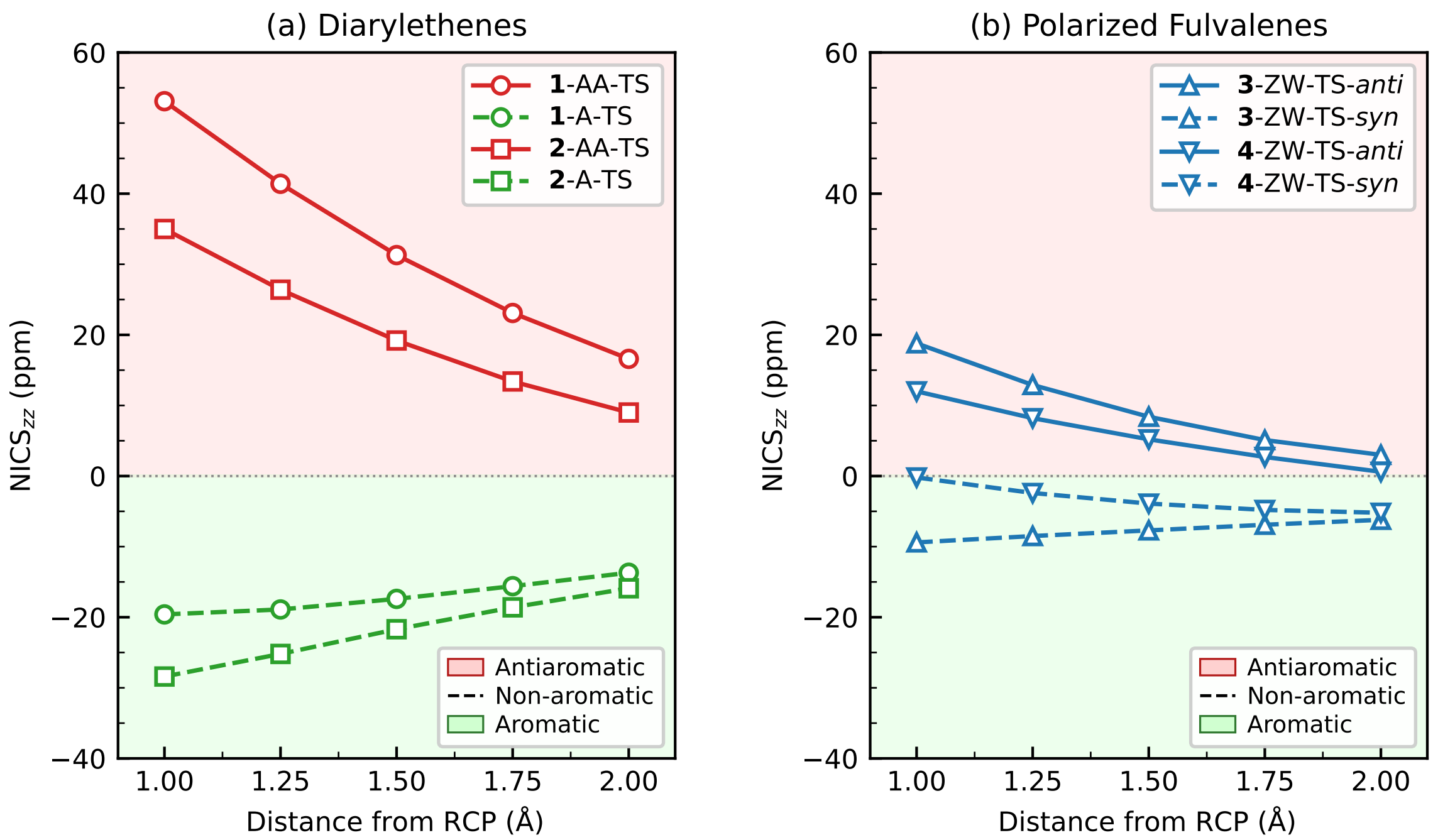

Figure S1: Distance dependence of the $NICS_{zz}$ component computed along the normal to plane of reactive hexatriene core, defined by the ring critical point (RCP) from atoms-in-molecules (AIM) analysis and the two central carbon atoms of the bridge connecting the two ring systems, for the transition states (TSs) of (a) diarylethenes **1** and **2** and (b) polarized fulvalenes **3** and **4**. Positive and negative $NICS_{zz}$ values indicate antiaromatic and aromatic character, respectively. The shaded regions denote the antiaromatic (red) and aromatic (green) sectors, and the dashed line at $NICS_{zz} = 0$ denotes the non-aromatic boundary.

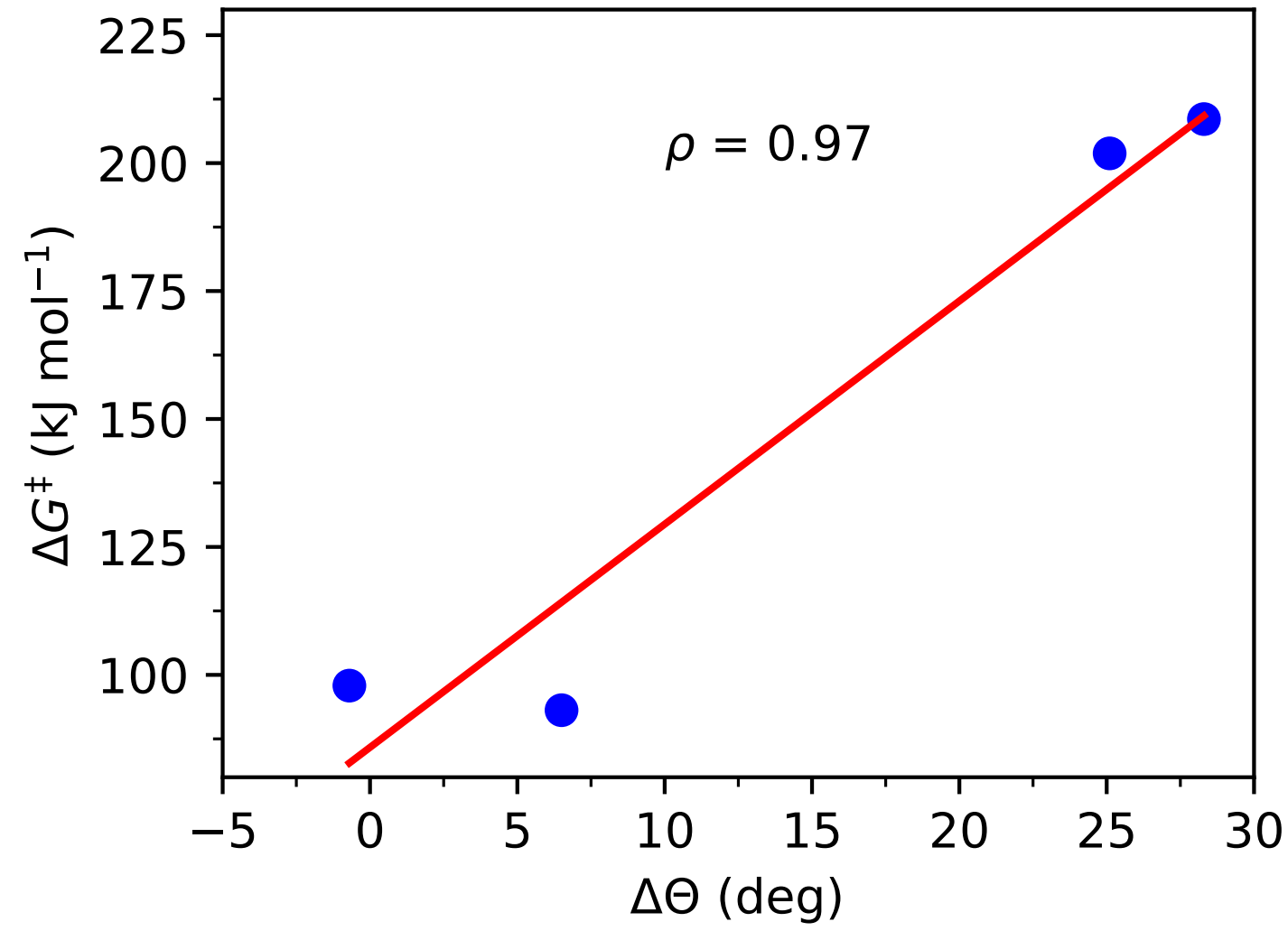

Figure S2: Electrocyclization free-energy barriers ($\Delta G^{\ddagger}$) as a function of changes in the Hilbert space mixing angle ($\Delta\Theta$) of antiaromatic transition states relative to associated reactants in conrotatory reaction pathways. Correlation coefficient (ρ) is shown.

References

(1) Zhao, Y.; Truhlar, D. G. The M06 Suite of Density Functionals for Main Group Thermochemistry, Thermochemical Kinetics, Noncovalent Interactions, Excited States, and Transition Elements: Two New Functionals and Systematic Testing of Four M06-Class Functionals and 12 Other Functionals. *Theor. Chem. Acc.* **2008**, *120*, 215–241.

(2) Bader, R. F. W. Atoms in molecules. *Acc. Chem. Res.* **1985**, *18*, 9–15.

(3) Lu, T.; Chen, F. Multiwfn: A multifunctional wavefunction analyzer. *J. Comput. Chem.* **2012**, *33*, 580–592.

(4) Frisch, M. J. et al. Gaussian 16 Revision C.01. 2016; Gaussian Inc. Wallingford CT.

(5) Roos, B. O.; Taylor, P. R.; Siegbahn, P. E. M. A complete active space SCF method (CASSCF) using a density matrix formulated super-CI approach. *Chem. Phys.* **1980**, *48*, 157–173.

(6) Siegbahn, P. E. M.; Almlöf, J.; Heiberg, A.; Roos, B. O. The complete active space SCF (CASSCF) method in a Newton–Raphson formulation with application to the HNO molecule. *J. Chem. Phys.* **1981**, *74*, 2384–2396.

(7) Li Manni, G. et al. The OpenMolcas Web: A Community-Driven Approach to Advancing Computational Chemistry. *J. Chem. Theory Comput.* **2023**, *19*, 6933–6991.

(8) Sun, Q. et al. Recent developments in the PySCF program package. *J. Chem. Phys.* **2020**, *153*, 024109.

Cartesian Coordinates

All geometries are reported in Cartesian coordinates (Å) and correspond to optimized structures at the levels of theory specified above.

1o

All frequencies are real.

C	0.766950	0.669597	-0.010396
C	0.766754	-0.669809	0.011509
C	2.042654	1.481056	0.003296
C	2.042370	-1.481499	-0.001342
C	3.282107	0.678287	0.291164
C	3.281541	-0.679128	-0.291300
H	2.142942	-1.961668	0.980055
H	2.142756	1.963107	-0.977220
C	-0.469607	-1.480292	0.091841
C	-1.452209	-1.302236	1.023520
C	-0.469163	1.480405	-0.091376
C	-1.971219	3.152260	0.503965
C	-1.451369	1.302517	-1.023484
C	-1.971892	-3.151605	-0.504442
C	-0.775882	-2.567068	-0.783942
C	-0.775490	2.567419	0.784099
S	-2.732558	2.410528	-0.832523
S	-2.733495	-2.410043	0.831994
H	-2.441933	3.977948	1.011800
H	-2.442630	-3.977093	-1.012581
H	-1.483355	0.553386	-1.798636
H	-1.484347	-0.553136	1.798698

C	4.290536	1.144461	1.019034
C	4.288648	-1.145480	-1.020880
H	4.266180	2.141910	1.441350
H	5.163245	0.535685	1.217775
H	5.161068	-0.536813	-1.221220
H	4.263454	-2.142978	-1.443026
H	1.950357	2.294409	0.726263
H	1.949700	-2.296173	-0.722742
H	-0.139421	2.881477	1.599707
H	-0.139552	-2.881134	-1.599350

1-AA-TS

This structure has one imaginary frequency.

C	0.891325	0.665963	-0.116509
C	0.891334	-0.665977	0.116530
C	2.158414	1.472398	-0.228655
C	2.158434	-1.472397	0.228679
C	3.394426	0.716297	0.181852
C	3.394433	-0.716292	-0.181853
H	2.264410	-1.799140	1.270699
H	2.264374	1.799165	-1.270669
C	-0.380644	-1.385533	0.256990
C	-1.579399	-0.670239	0.645857
C	-0.380660	1.385511	-0.256966
C	-2.005463	2.980095	0.230197
C	-1.579428	0.670202	-0.645784
C	-2.005470	-2.980055	-0.230294

C	-0.672865	-2.644122	-0.276291
C	-0.672863	2.644151	0.276202
S	-2.953755	1.765552	-0.536019
S	-2.953736	-1.765565	0.536050
H	-1.593105	0.038971	-1.528650
H	-1.593049	-0.039030	1.528737
H	-2.469332	3.871902	0.618279
H	-2.469357	-3.871838	-0.618408
C	4.404355	-1.287492	-0.828254
C	4.404353	1.287503	0.828241
H	2.073515	-2.385189	-0.365144
H	2.073496	2.385177	0.365187
H	5.275068	-0.714783	-1.120598
H	4.383001	-2.338766	-1.089436
H	4.382991	2.338775	1.089432
H	5.275077	0.714802	1.120568
H	0.065548	3.287357	0.734235
H	0.065531	-3.287292	-0.734395

1-A-TS

This structure has one imaginary frequency.

C	0.852840	0.688303	0.015489
C	0.833742	-0.712809	0.040982
C	2.108623	1.345109	0.547075
C	2.090741	-1.383822	0.558085
C	3.369629	0.699915	0.015574
C	3.296959	-0.774891	-0.112858

H	2.059103	-2.455804	0.374446
H	2.116002	2.409647	0.320151
C	-0.358198	-1.438215	-0.045576
C	-1.493553	-0.990217	-0.807731
C	-0.322446	1.436600	-0.102277
C	-1.958703	2.866734	0.734709
C	-1.462693	0.984851	-0.854518
C	-2.020224	-2.792550	0.862983
H	-2.462046	3.561016	1.389337
H	-2.533786	-3.446341	1.550531
C	4.156929	-1.512221	-0.808463
C	4.449726	1.423153	-0.263184
H	-1.341613	0.942773	-1.924317
H	2.116976	1.250826	1.642251
H	2.167000	-1.233693	1.643544
H	4.438266	2.501515	-0.169031
H	5.377984	0.966702	-0.580364
H	4.044831	-2.586854	-0.871539
H	4.987160	-1.071226	-1.344222
H	-1.383175	-1.000805	-1.879446
S	-2.874650	1.950369	-0.409008
S	-2.925520	-1.898633	-0.306988
C	-0.701996	-2.504901	0.853682
H	0.000069	-2.938420	1.552140
C	-0.648250	2.545424	0.751542
H	0.057196	2.990105	1.439385

1c-*anti*

All frequencies are real.

C	-0.883411	0.637993	-0.467919
C	-0.777280	-0.820528	-0.453312
C	-2.226812	1.183125	-0.869369
C	-2.081332	-1.574970	-0.549073
C	-3.351534	0.544217	-0.075547
C	-3.133008	-0.879044	0.282747
H	-1.963413	-2.606827	-0.224469
H	-2.272411	2.265663	-0.772165
C	0.432366	-1.408498	-0.376117
C	1.681241	-0.544713	-0.385571
C	0.170030	1.393041	-0.099056
C	1.577877	3.238456	0.136198
C	1.434130	0.714008	0.399121
C	2.068775	-3.030112	-0.009846
C	0.780735	-2.811789	-0.308356
C	0.316846	2.832465	-0.064936
S	2.760798	1.964552	0.302050
S	3.036245	-1.597725	0.238174
H	1.912806	4.264178	0.183551
H	2.537560	-3.996408	0.104509
C	-3.739821	-1.485190	1.298134
C	-4.457861	1.217836	0.223235
H	1.303371	0.457682	1.459524
H	1.925302	-0.280512	-1.423856
H	-2.389117	0.953335	-1.930057
H	-2.406957	-1.596005	-1.596708

H	-4.563445	2.263090	-0.038932
H	-5.294242	0.746373	0.722292
H	-3.542916	-2.525197	1.525002
H	-4.435318	-0.961411	1.940815
H	0.070435	-3.612850	-0.453206
H	-0.499981	3.521875	-0.221895

1c-*syn*

All frequencies are real.

C	0.797236	0.663750	0.218129
C	0.836520	-0.787636	0.011234
C	2.015913	1.252972	0.879522
C	2.140426	-1.456651	0.376761
C	3.304862	0.765565	0.248541
C	3.285491	-0.652037	-0.190344
H	2.174876	-2.478831	0.006302
H	1.988426	2.340619	0.867888
C	-0.278475	-1.475836	-0.292245
C	-1.567513	-0.806750	-0.664732
C	-0.332423	1.352076	-0.021667
C	-2.008682	2.957485	0.212230
C	-1.447100	0.706813	-0.810565
C	-1.826435	-3.012677	0.488719
H	-2.509973	3.867278	0.507707
H	-2.248843	-3.915489	0.904622
C	4.138467	-1.170222	-1.068395
C	4.370196	1.555182	0.154695

H	-1.201972	0.880228	-1.862959
H	2.021021	0.947321	1.934054
H	2.229623	-1.498007	1.469547
H	4.327343	2.589840	0.470637
H	5.317332	1.194064	-0.223823
H	4.074765	-2.210984	-1.359029
H	4.912975	-0.574387	-1.533151
H	-1.880912	-1.208198	-1.630892
S	-2.959429	1.680475	-0.501248
S	-2.804731	-1.549833	0.487341
C	-0.574411	-2.870768	0.034966
H	0.158712	-3.664093	0.060880
C	-0.705849	2.688576	0.382133
H	-0.026143	3.381316	0.856832

2o

All frequencies are real.

C	0.706282	0.700428	0.007886
C	0.706272	-0.700461	-0.007844
C	1.933233	1.365365	0.031101
C	1.933209	-1.365421	-0.031100
C	3.149338	0.700007	0.019743
C	3.149327	-0.700086	-0.019778
H	1.929356	-2.449957	-0.030018
H	1.929423	2.449901	0.030001
C	-0.526450	-1.518242	0.028872
C	-1.571338	-1.330555	0.888434

C	-0.526424	1.518238	-0.028813
C	-1.940716	3.256853	0.590006
C	-1.571466	1.330419	-0.888158
C	-1.940856	-3.256651	-0.590108
C	-0.748945	-2.643579	-0.821917
C	-0.748790	2.643750	0.821780
S	-2.804154	2.486276	-0.665752
S	-2.804227	-2.486255	0.665734
H	-2.356764	4.114217	1.092963
H	-2.357043	-4.113909	-1.093142
H	-1.675060	0.557076	-1.632600
H	-1.674751	-0.557330	1.633014
C	4.442756	1.463973	0.039008
H	5.052915	1.231325	-0.836634
H	4.262884	2.537565	0.051782
H	5.038838	1.208373	0.918155
C	4.442730	-1.464077	-0.039046
H	5.038909	-1.208357	-0.918090
H	5.052803	-1.231573	0.836697
H	4.262836	-2.537663	-0.051996
H	-0.049493	2.960075	1.583061
H	-0.049727	-2.959846	-1.583296

2-AA-TS

This structure has one imaginary frequency.

C	0.848885	0.697523	-0.110989
C	0.848880	-0.697515	0.111004

C	2.065248	1.355600	-0.225861
C	2.065242	-1.355599	0.225857
C	3.284387	0.689081	-0.114553
C	3.284382	-0.689087	0.114537
H	2.064705	-2.420900	0.429500
H	2.064715	2.420898	-0.429518
C	-0.435284	-1.402946	0.176225
C	-1.637518	-0.694066	0.578810
C	-0.435278	1.402961	-0.176206
C	-2.055064	2.944351	0.464313
C	-1.637502	0.694098	-0.578799
C	-2.055042	-2.944334	-0.464356
C	-0.720777	-2.611970	-0.459064
C	-0.720795	2.611997	0.459049
S	-3.015270	1.787085	-0.374148
S	-3.015265	-1.787122	0.374159
H	-1.668781	0.150646	-1.518757
H	-1.668732	-0.150650	1.518793
H	-2.515470	3.796305	0.937040
H	-2.515399	-3.796302	-0.937113
C	4.578149	-1.442109	0.242538
H	4.398563	-2.498886	0.432964
H	5.176812	-1.358285	-0.667603
H	5.186975	-1.048352	1.059710
C	4.578156	1.442104	-0.242531
H	5.176646	1.358573	0.667754
H	5.187154	1.048128	-1.059466

H	4.398568	2.498819	-0.433297
H	0.033175	3.207909	0.955707
H	0.033201	-3.207852	-0.955745

2-A-TS

This structure has one imaginary frequency.

C	-0.797000	0.718316	-0.325622
C	-0.797144	-0.718150	-0.326115
C	0.417381	1.425044	-0.226367
C	0.417084	-1.425191	-0.226872
C	1.646896	0.911747	-0.807728
C	1.647016	-0.911979	-0.807487
C	0.653211	2.492513	0.689786
C	0.652276	-2.492916	0.689157
H	-0.134838	2.942815	1.277308
H	-0.136185	-2.943280	1.276079
C	1.963864	2.763317	0.883991
C	1.962786	-2.763751	0.884263
S	3.016451	1.826888	-0.117150
S	3.016110	-1.827079	-0.115860
C	-2.046455	1.380609	-0.154851
C	-2.046759	-1.380299	-0.155905
C	-3.230438	-0.716560	-0.035568
C	-3.230273	0.717031	-0.035035
H	-2.047047	2.464820	-0.164055
H	-2.047573	-2.464501	-0.165868
H	1.681294	-0.992909	-1.885115

H	2.380803	-3.424149	1.627881
H	1.680342	0.992440	-1.885402
H	2.382439	3.423618	1.627380
C	-4.527866	-1.462712	0.085878
H	-4.360006	-2.538235	0.075952
H	-5.046593	-1.206441	1.012407
H	-5.205121	-1.216288	-0.735129
C	-4.527552	1.463353	0.086873
H	-5.204489	1.218025	-0.734757
H	-5.046683	1.206062	1.012882
H	-4.359492	2.538859	0.078217

2c-*anti*

All frequencies are real.

C	0.787225	0.731799	0.020395
C	0.787236	-0.731805	-0.020429
C	2.064755	1.394289	0.126710
C	2.064774	-1.394283	-0.126715
C	3.235386	0.726257	0.079922
C	3.235397	-0.726239	-0.079904
H	2.075960	-2.472896	-0.226312
H	2.075929	2.472901	0.226320
C	-0.391825	-1.410410	0.097058
C	-1.665934	-0.644488	0.382900
C	-0.391845	1.410388	-0.097121
C	-1.952280	3.138831	-0.022512
C	-1.665933	0.644455	-0.382924

C	-1.952273	-3.138830	0.022470
C	-0.645593	-2.825448	0.058520
C	-0.645608	2.825428	-0.058624
S	-3.042811	1.779405	-0.005969
S	-3.042775	-1.779405	0.006053
H	-2.364020	4.136467	0.013737
H	-2.364040	-4.136453	-0.013819
C	4.546963	-1.449649	-0.177963
C	4.546941	1.449682	0.178015
H	-1.710151	0.421303	-1.461087
H	-1.710031	-0.421283	1.461061
H	5.162209	1.280040	-0.708432
H	4.390253	2.521735	0.283569
H	5.126365	1.103867	1.037148
H	5.162204	-1.280002	0.708503
H	4.390288	-2.521704	-0.283523
H	5.126407	-1.103827	-1.037079
H	0.131833	3.575031	-0.030932
H	0.131843	-3.575051	0.030724

2c-*syn*

All frequencies are real.

C	0.700714	-0.770278	-0.195328
C	0.809852	0.673833	-0.443377
C	-0.526381	-1.363537	-0.178736
C	-0.309187	1.450849	-0.518268
C	-1.694584	-0.637597	-0.787232

C	-1.685982	0.880191	-0.624448
C	-0.929090	-2.633727	0.350546
C	-0.428897	2.845129	-0.113997
H	-0.240361	-3.354221	0.767266
H	0.359777	3.578311	-0.200433
C	-2.263136	-2.811092	0.380982
C	-1.560175	3.072849	0.570100
S	-3.209272	-1.500910	-0.262839
S	-2.639339	1.697139	0.735723
C	1.910577	-1.483278	0.133784
C	2.133554	1.253674	-0.384720
C	3.238156	0.534953	-0.103218
C	3.122702	-0.894837	0.174522
H	1.834569	-2.548031	0.319780
H	2.231304	2.312610	-0.591157
H	-2.159200	1.302233	-1.513571
H	-1.814730	3.992253	1.076266
H	-1.613305	-0.803785	-1.867239
H	-2.778108	-3.664356	0.796503
C	4.594117	1.177460	-0.067697
H	4.524925	2.240474	-0.291436
H	5.060234	1.063990	0.913579
H	5.268647	0.718518	-0.794125
C	4.361017	-1.681890	0.490278
H	5.081168	-1.637281	-0.329969
H	4.864933	-1.289294	1.376429
H	4.118386	-2.727292	0.672817

3o

All frequencies are real.

C	2.930363	-1.664621	0.204033
C	3.734836	-0.738127	-0.343780
C	3.488414	0.680707	-0.450651
C	1.638763	-1.452504	0.814874
C	2.351960	1.371632	-0.254022
C	0.829441	-0.379912	0.766089
C	1.016077	0.908951	0.109797
H	3.296389	-2.684546	0.232739
H	4.694054	-1.073138	-0.721061
H	4.345296	1.266999	-0.763922
H	1.258933	-2.299146	1.376238
H	2.413031	2.442185	-0.423533
H	-0.114283	-0.473239	1.291576
C	-0.010688	1.791956	-0.035896
C	-1.413696	1.606739	0.249343
H	0.258661	2.797594	-0.344877
C	-2.193967	0.528913	0.013623
C	-3.606499	0.426622	0.394522
C	-1.874355	-0.706998	-0.702419
C	-4.071400	-0.762318	-0.033321
C	-2.987569	-1.466709	-0.730140
H	-5.072239	-1.146360	0.096171
H	-3.088738	-2.431352	-1.205696
H	-1.915610	2.478246	0.664926
H	-4.144193	1.187339	0.940344

H	-0.921374	-0.930574	-1.155209

3-ZW-TS-*anti*

This structure has one imaginary frequency.

C	-2.323707	-1.694754	-0.354437
C	-3.274124	-0.851877	0.228730
C	-3.154650	0.509298	0.427721
C	-1.056438	-1.396292	-0.802784
C	-2.069177	1.357284	0.211507
C	-0.311717	-0.207059	-0.681501
C	-0.770481	1.078806	-0.215427
H	-2.645949	-2.717525	-0.520103
H	-4.221088	-1.302010	0.498073
H	-4.034528	0.999273	0.831414
H	-0.529994	-2.197211	-1.307823
H	-2.240519	2.395068	0.476172
H	0.574497	-0.172254	-1.302454
C	0.194741	2.148616	-0.245820
C	1.534004	1.922129	-0.117875
H	-0.180667	3.158204	-0.362565
C	2.048799	0.654573	0.265004
C	3.138287	0.000357	-0.338442
C	1.281209	-0.344182	0.966087
C	3.046046	-1.367735	-0.047274
C	1.931656	-1.572933	0.780292
H	3.730012	-2.130987	-0.386976
H	1.652097	-2.509801	1.239532

H	2.226258	2.731477	-0.331475
H	3.856317	0.466151	-0.998255
H	0.526883	-0.137774	1.709771

3-ZW-TS-*syn*

This structure has one imaginary frequency.

C	-2.225236	-1.770526	-0.021862
C	-3.098011	-0.919332	-0.719135
C	-3.081308	0.452727	-0.771742
C	-1.185800	-1.421246	0.805254
C	-2.108744	1.337490	-0.278121
C	-0.592040	-0.170215	1.034959
C	-0.922042	1.087127	0.381484
H	-2.463471	-2.826632	-0.064168
H	-3.912650	-1.406987	-1.242257
H	-3.893057	0.919349	-1.317093
H	-0.746360	-2.233359	1.375201
H	-2.275602	2.382955	-0.521491
C	0.050299	2.135073	0.421589
C	1.385666	1.892904	0.250127
H	-0.304710	3.150896	0.294648
C	2.017754	0.629143	0.117467
C	2.857784	0.322864	-0.955096
C	1.705312	-0.634422	0.793657
C	3.019352	-1.074720	-1.004015
C	2.344524	-1.655104	0.067960
H	3.586711	-1.608113	-1.753708

H	2.398068	-2.692542	0.362160
H	1.985745	2.752822	-0.036006
H	3.190438	1.028151	-1.702828
H	-0.155528	-0.025718	2.008466
H	1.585353	-0.711400	1.861910

3c-*anti*

All frequencies are real.

C	-1.174430	-0.564732	0.389634
C	-2.051851	0.615125	0.103725
C	-1.520401	1.928030	0.368860
C	-0.187342	2.098406	0.426139
C	0.763466	1.041685	0.098991
C	0.286715	-0.395215	-0.048564
C	-1.962166	-1.717615	-0.154462
C	-3.152548	-1.266622	-0.582298
C	-3.218857	0.185962	-0.416516
H	-2.189378	2.772565	0.482305
H	0.224367	3.085610	0.601218
H	-1.624117	-2.743763	-0.141834
H	-4.058619	0.804861	-0.699794
H	-3.950396	-1.871174	-0.989816
C	2.020797	1.383869	-0.263297
C	1.167789	-1.307298	0.767426
H	0.693334	-1.841482	1.584810
H	2.265946	2.441459	-0.250466
C	2.463278	-1.523173	0.521339

C	3.058248	0.512584	-0.754441
H	2.981420	-2.278901	1.102359
C	3.255119	-0.785068	-0.434885
H	3.808203	0.987681	-1.378006
H	4.145924	-1.269108	-0.819076
H	0.386055	-0.663424	-1.108572
H	-1.169638	-0.659964	1.486969

3c-*syn*

All frequencies are real.

C	-1.870443	-1.576428	-0.892954
C	-3.026850	-1.122932	-0.160105
C	-3.191473	0.116708	0.373336
C	-0.615700	-1.156783	-0.664656
C	-2.249428	1.197859	0.286691
C	-0.308098	-0.305438	0.529159
C	-0.903258	1.055528	0.228054
H	-2.049816	-2.284633	-1.694669
H	-3.890849	-1.778499	-0.158901
H	-4.176349	0.360435	0.757065
H	0.198916	-1.435798	-1.325502
H	-2.664390	2.197568	0.200518
C	-0.037662	2.145167	-0.191854
C	1.281009	1.979917	-0.409931
H	-0.513912	3.091274	-0.421657
C	1.893890	0.711537	-0.103351
C	3.005052	0.100719	-0.564409

C	1.184485	-0.208402	0.847391
C	3.067918	-1.249870	-0.006347
C	2.006563	-1.460140	0.789881
H	3.858003	-1.958356	-0.211473
H	1.787792	-2.356204	1.352251
H	1.873890	2.775569	-0.844318
H	3.721990	0.518494	-1.257603
H	-0.844090	-0.721538	1.387649
H	1.282778	0.227034	1.851228

4o

All frequencies are real.

C	0.897208	0.563413	-0.058056
C	-0.090530	1.491190	-0.060716
C	-1.499583	1.305638	-0.286301
H	0.214434	2.515595	0.140209
C	-2.153601	0.164330	-0.020131
C	-2.212021	-1.162502	0.481985
C	-3.338479	-0.611377	0.082562
H	-2.083567	2.162147	-0.596558
C	0.857245	-0.840923	-0.452486
C	2.097015	-1.356515	-0.283502
H	0.008982	-1.343836	-0.888693
C	2.981856	-0.315577	0.231186
H	2.404273	-2.365174	-0.519356
C	2.275248	0.830185	0.345529
H	4.026774	-0.453024	0.465966

H	2.629047	1.787150	0.699858
H	-1.685374	-2.009995	0.885053
H	-4.400723	-0.700040	-0.066897

4-ZW-TS-*anti*

This structure has one imaginary frequency.

C	0.607075	0.859084	0.303279
C	-0.516861	1.729525	0.176302
C	-1.796341	1.234331	0.091782
H	-0.362902	2.804672	0.141289
C	-1.857571	-0.150253	-0.158516
C	-0.903718	-1.087859	-0.584391
C	-2.184341	-1.460127	-0.222548
H	-2.680624	1.849282	0.159140
C	0.568980	-0.441281	0.920428
C	1.721217	-1.134246	0.508084
H	-0.050923	-0.703002	1.765368
C	2.405245	-0.347137	-0.426311
H	2.034951	-2.102519	0.870209
C	1.746617	0.888463	-0.522370
H	3.303746	-0.624939	-0.955572
H	2.012383	1.699421	-1.186222
H	-2.892554	-2.240236	-0.006725
H	-0.105904	-1.225674	-1.301919

4-ZW-TS-*syn*

This structure has one imaginary frequency.

C	-0.689781	0.709759	-0.307698
C	0.377576	1.630675	-0.300478
C	1.722137	1.349954	-0.170231
H	0.099568	2.671432	-0.156423
C	2.014009	0.000005	0.044037
C	1.484865	-1.277659	-0.241264
C	2.358031	-1.063649	0.802244
H	2.415759	2.123440	0.123103
C	-0.689039	-0.710589	-0.675580
C	-1.886591	-1.252823	-0.168645
H	-0.292988	-1.039149	-1.623130
C	-2.580020	-0.258743	0.520453
H	-2.243994	-2.254016	-0.359901
C	-1.874805	0.947440	0.405146
H	-3.509719	-0.390133	1.054376
H	-2.124062	1.879712	0.892517
H	2.883162	-1.452707	1.657830
H	1.353983	-1.984793	-1.036278

4c-*anti*

All frequencies are real.

C	-0.579483	0.790796	-0.151182
C	0.525286	1.731994	-0.081261
C	1.800844	1.312618	0.057785
H	0.301566	2.791560	-0.130747
C	1.964383	-0.113412	0.156615
C	0.882343	-1.100320	0.464637

C	2.215846	-1.340263	-0.172179
H	2.635743	1.999084	0.080700
C	-0.351789	-0.698827	-0.332897
C	-1.706586	-1.274109	-0.065450
H	-0.132042	-0.861883	-1.399940
C	-2.589069	-0.276725	0.114513
H	-1.928361	-2.331119	-0.090988
C	-1.894731	1.007480	0.055279
H	-3.651465	-0.389001	0.277549
H	-2.366196	1.971755	0.191033
H	2.836135	-2.040400	-0.704878
H	0.702352	-1.375388	1.502107

4c-*syn*

All frequencies are real.

C	-0.433358	0.862309	-0.190266
C	0.784406	1.665102	-0.223249
C	1.976075	1.071632	-0.033958
H	0.712122	2.743017	-0.306417
C	1.874950	-0.358174	0.164953
C	0.871773	-1.290921	-0.450138
C	1.549476	-1.389314	0.874354
H	2.907011	1.617304	0.026407
C	-0.460995	-0.579514	-0.676164
C	-1.791303	-1.070866	-0.184943
C	-2.460121	-0.052663	0.376438
H	-2.164111	-2.069444	-0.359759

C	-1.619316	1.145107	0.381786
H	-3.466806	-0.092405	0.767642
H	-1.896486	2.090328	0.827875
H	1.534316	-1.852595	1.845912
H	1.189597	-1.937223	-1.263085
H	-0.565162	-0.515175	-1.771451
